# Genome wide methylation profiling of selected matched soft tissue sarcomas identifies methylation changes in metastatic and recurrent disease

**DOI:** 10.1038/s41598-020-79648-6

**Published:** 2021-01-12

**Authors:** Ana Cristina Vargas, Lesley-Ann Gray, Christine L. White, Fiona M. Maclean, Peter Grimison, Nima Mesbah Ardakani, Fiona Bonar, Elizabeth M. Algar, Alison L. Cheah, Peter Russell, Annabelle Mahar, Anthony J. Gill

**Affiliations:** 1 Department of Anatomical Pathology, Douglass Hanly Moir Pathology, Macquarie Park, NSW 2113 Australia; 2 Cancer Diagnosis and Pathology Group, Kolling Institute of Medical Research, Royal North Shore Hospital, St Leonards, NSW 2065 Australia; 3 Medical School, University of Sydney, Sydney, NSW 2006 Australia; 4 Australian Genome Research Facility Ltd, Victorian Comprehensive Cancer Centre, Melbourne, VIC 3000 Australia; 5 Genetics and Molecular Pathology Laboratory, Centre for Cancer Research, Hudson Institute of Medical Research, Clayton, VIC 3168 Australia; 6 Monash University, Clayton, VIC Australia; 7 Department of Clinical Medicine, Faculty of Medicine and Health Sciences, Macquarie University, Sydney, NSW Australia; 8 Department of Medical Oncology, Chris O’Brien Lifehouse, Camperdown, NSW 2050 Australia; 9 Department of Anatomical Pathology, PathWest Laboratory Medicine, QEII Medical Centre, Perth, WA Australia; 10 School of Pathology and Laboratory Medicine, University of Western Australia, Perth, WA Australia; 11 Department of Tissue Pathology and Diagnostic Oncology, Royal Prince Alfred Hospital, Camperdown, NSW 2050 Australia; 12 NSW Health Pathology, Department of Anatomical Pathology, Royal North Shore Hospital, Sydney, NSW 2065 Australia

**Keywords:** Cancer, Oncology

## Abstract

In this study we used the Illumina Infinium Methylation array to investigate in a cohort of matched archival human tissue samples (n = 32) from 14 individuals with soft tissue sarcomas if genome-wide methylation changes occur during metastatic and recurrent (Met/Rec) disease. A range of sarcoma types were selected for this study: leiomyosarcoma (LMS), myxofibrosarcoma (MFS), rhabdomyosarcoma (RMS) and synovial sarcoma (SS). We identified differential methylation in all Met/Rec matched samples, demonstrating that epigenomic differences develop during the clonal evolution of sarcomas. Differentially methylated regions and genes were detected, not been previously implicated in sarcoma progression, including at *PTPRN2* and *DAXX* in LMS, *WT1-AS* and *TNXB* in SS, *VENTX* and *NTRK3* in pleomorphic RMS and *MEST* and the *C14MC* / miR-379/miR-656 in MFS. Our overall findings indicate the presence of objective epigenetic differences across primary and Met/Rec human tissue samples not previously reported.

## Introduction

DNA methylation, a specific type of epigenetic modification, leads to changes in gene expression without modifications of the DNA sequence. It involves the addition of a methyl group to the carbon-5 position at cytosine residues on cytosine-phosphate-guanine (CpG) dinucleotides^1^. Clusters rich in CpG sites, known as CpG islands, are involved in the control of transcription and gene expression, wherein unmethylated CpG islands allow transcription, whilst hypermethylated promoter regions repress gene expression^1–4^. Methylation alterations have been implicated in transformation of mesenchymal stem cells during sarcomagenesis^5,6^. Moreover, comprehensive DNA methylation profiling in soft tissue sarcomas (STSs) has previously shown that such epigenetic modifications are crucial in the biology and prognosis of several sarcoma types including its value as a tool to refine tumour classification^7–15^ (Ref added). Nonetheless, the contribution of genome-wide DNA methylation changes during progression of STSs has not been widely explored in matched metastatic/recurrent (Met/Rec) sarcoma samples. The Illumina Infinium MethylationEPIC BeadChip^16–18^ array interrogates over 850,000 methylation sites including CpG islands and has been previously validated on restored DNA from formalin-fixed paraffin-embedded (FFPE) tissue^12–14,19–22^ (References added). High correlation has been observed in these studies between matched fresh and FFPE tissue samples despite the lower quality of FFPE tissue. Thus it is now possible to interrogate archival FFPE clinical samples from patients who present with metastatic or recurrent disease during the natural course of sarcoma evolution.

Methylation status becomes a more attractive target of analysis given genome-wide studies have not revealed specific genetic drivers of sarcoma metastasis (Reviewed in^23^). In the only study available with comprehensive genomic (but no epigenomic) data on specific sub-types of matched metastatic/recurrent sarcomas, Hofvander et al.^24^, demonstrated no major changes occurred at the genetic level during clonal evolution. The authors hypothesized that in the absence of obvious genomic differences, it is possible that epigenetic modifications play a major role in the metastatic cascade of sarcomas. DNA methylation changes were not identified to occur in a single study available of matched primary and recurrent well-differentiated liposarcomas^25^ but methylation analysis on other paired sarcoma types has not been published to date.

Leiomyosarcoma (LMS) and Myxofibrosarcoma (MFS) are examples of some of the most frequent sporadic sarcomas in the adult population^26^. These are typified by a complex karyotype with no recurrent genomic alterations present. Pleomorphic rhabdomyosarcoma (Ple-RMS), whilst rare, is an aggressive sarcoma with poor prognosis and is also characterised by a complex karyotype^20^. In contrast Synovial sarcoma (SS) and Embryonal rhabdomyosarcoma (Emb-RMS) present in younger individuals, with the latter more commonly seen in a paediatric population. SS is characterised by the t(X;18)(p11.2;q11.2) translocation^27^ and Emb-RMS (sporadic) frequently harbours recurrent genetic alterations including LOH at 11p15 and polysomy 8^26^. A shared issue across sarcomas regardless of their molecular signature and histological type, is a lack of biological predictors to determine which patients will remain free of disease and who will progress to develop advanced incurable tumours due to recurrence and/or metastatic disease. Furthermore, for those patients with refractory disease, there are no specific therapeutic targets available. In this study we sought to investigate in a limited number of FFPE sarcoma samples (n = 32) from 14 individuals (LMS: n = 7; MFS: n = 4; RMS: n = 2; and SS: n = 1) whether methylation changes at a genome-wide level arise in metastatic or recurrent disease. Our aim is to discover whether such changes play a role in the biology of recurrent/metastatic behaviour, which may enable the identification of predictive/prognostic biomarkers and/or potential therapeutic targets. Although our cohort is limited, our study is the first to address the contribution of methylation in progression of selected sarcoma types on paired human tissue samples.

## Materials and methods

### Sample selection and histopathological assessment

This study was approved by the Northern Sydney Local Health District (NSLHD) Human Research Ethics Committee (HREC) reference 1312-417 M. Informed consent was not obtained as this is a retrospective cohort of de-identified archival tissue samples, which is compliant with HREC for de-identified human tissue samples. All methods included in this study were performed in accordance with the relevant guidelines and regulations. A retrospective database search was performed in the archives of Douglass Hanly Moir (DHM) Pathology laboratory to identify FFPE tissue blocks derived from surgical resections (biopsies excluded) of patients with selected sarcoma types (LMS, MFS, SS & RMS), who developed metastatic (Mets) and/or recurrent (Rec) tumours within a 5-year period and whose residual archival tumour blocks were adequate for further molecular analysis (i.e. viable non-necrotic tumour with adequate formalin fixation). All the cases were originally diagnosed in the clinical setting by pathologists with expertise in sarcomas and/or gynaecological pathology following international guidelines as per the World Health Organization (WHO) classification and independently reviewed for this study to ensure diagnoses were in keeping with the current WHO 2020 classifications of soft tissue tumours^26^. The tumours were graded according to the French Federation of Cancer Centers Sarcoma Group (FNCLCC) system. Histopathological criteria for diagnosis included the use of immunohistochemical stains (IHC) and fluorescent in situ hybridization (FISH) depending on the tumour entity (Suppl. Methods and Tables 1, 2). A second retrospective histological review was performed to determine whether there was morphological variation between paired samples in any of the following features: FNCLCC grade, tumour cell pleomorphism, cellularity, change in the morphological appearances of the cells (i.e. shifting from spindle to epithelioid or vice versa) and/or acquisition of pleomorphic tumour giant cells or other cellular elements not originally identified. Estimation of the content of tumour-infiltrating lymphocytes (TILs)/plasma cells assessed on haematoxylin and eosin (H&E)-stained sections was also performed using a threshold of 10% to determine presence versus absence of increased TILs.

**Table 1:** Clinical and pathological features of matched leiomyosarcoma samples.

Paired set No	Label ID	Age/ gender	Anatomic site	Sample type	Year of Dx	Diagnosis	Immunophenotype and further analyses	Previous treatment	Additional Mets/Rec not analysed	Cell morphology; morphological variation	TILs (> 10%)
Set 1	LM120	F61	Rigth psoas	Primary	Aug, 2014	Pleomorphic leiomyosarcoma, 50 mm, Gde 3	**Pos** for Desmin, SMA, Caldesmon, Actin & PR. **Neg** for S100, DOG1, CD34, CD117 & ER. Ki-67—up to 80%	No prior treatment; clinical diagnosis of leiomyoma	Further mets to lung and pleura in 2017 and 2019	Spindle and pleomoprhic	No
	LM106		Lung	Mets	March, 2017					Spindle and pleomoprhic	No
Set 2	LM020	F56	Uterus	Primary	Nov, 2013	Leiomyosarcoma, 90 mm, Gde 3	**Pos** for Caldesmon, Myogenin, Actin & CD10. **Neg** for for Myoglobin, Pan CK, HMB45, ER & PR	No prior therapy; clinical dx of leiomyoma	Last FU in 2014. Also developed pancreatic cancer	Predominantly spindle	No
	LM117		Lung	Mets	Dec, 2014			Chemo-radiotherapy (Specimen post-induction chemotherapty)		Epithelioid and acquired pleomorphic giant cells	No
Set 3	LM028	F64	Uterus	Primary	Nov, 2012	Epithelioid leiomyosarcoma, 95 mm	**Pos** for SMA, Desmin (weak), ER & PR. Neg for CD31, CD34, HMB45 & CD10	No prior therapy; clinically leiomyoma	No further follow up (2013)	High number of giant cells	No
	LM024		Vagina	Mets	Feb, 2013			Radiotherapy		More cellular and epithelioid	No
Set 4	LM051	F32	Uterus	Primary	March, 2010	Leiomyosarcoma, 124 mm, low grade	**Pos** for Desmin, ER & PR	No prior therapy; clinically leiomyoma; rapidly grow	No development of further disease; FU till 2017	Epithelioid and spindled; few giant cells	No
	LM116		Lung	Mets	Feb, 2015					Spindled and cellular	No
Set 5	LM015	F59	Colon peritoneum	Mets 1	July, 2012	Mets leiomyosarcoma, primary uterine; 124 mm, low grade	**Pos** for Desmin and SMA	Prior chemotherapy	Original diagnosis in 2005—primary tumour in the omentum	Bland spindle cells	No
	LM119		Colon + pelvis	Mets 2	Nov, 2014			Chemo-radiotherapy		Increased cellularity & atypia	No
	LM080		Colon/bladder	Mets 3	Nov, 2016			Posterior exenteration		Aquired pleomorphic giant cells	No
Set 6	LM078	F50	Lung	Mets 1	Dec, 2016	Metastatic leiomyosarcoma from primary uterine, high gde	**Pos** for Actin, SMA, Desmin, Calponin & ER		Original dx in 2014. Four additional mets between 2016–2020 to lung & chest wall. Alive	Oval and epithelioid with pleom giant cells	No
	LM085		Lung	Mets 2	Aug, 2017					Same histology; no change	No
Set 7	LM019	F43	Kidney	Primary	Nov, 2013	Leiomyosarcoma, 110 mm, Grade 2	**Pos** for Desmin & SMA. **Neg** for Actin, EMA, Pan-CK, CK7, CK19, Melan A, HMB45, PAX8, CD117, Myogenin, CD34, CD31 & CD99	No prior therapy	Primary from kidney; localised at presentation	Spindle, hypercellular	No
	LM118		Omentum	Mets	May, 2014		**aCGH**: Gains in 6q, 8p, 8q, 9p, 11q & 12q; Amp in 11q & 12q; Loss in 9p	Chemotherapy	Mets to soft tissue (deltoid) in 2014 and peritoneum	Highly pleomorphic, significant change	No

Mets: Metastasis; Gde: Grade (FNCLCC system); Pos: Positive; Neg: Negative; SMA: Smooth Muscle Actin; PR; Progesterone Receptor, ER: Estrogen Receptor; TILs: Tumour-infiltrating lymphocytes; Dx: diagnosis; FU: Follow-up; aCGH: array comparative genomic hybridization; Amp: Amplification; FISH: Fluorescent in-situ hybridization (break-apart probes).

**Table 2:** Clinical and pathological features of matched myxofibrosarcoma, synovial sarcoma and rhabdomyosarcoma samples.

Paired set No	Label ID	Age/ gender	Anatomic site	Sample type	Year of Dx	Diagnosis	Immunophenotype and further analyses	Previous treatment	Additional Mets/Rec not analysed	Cell morphology; morphological variation	TILs (> 10%)
Set 1	MFS-77	F76	Tibia	Primary	Feb, 2012	Myxofibrosarcoma, 38 mm; myoid diff; gde 3; eroding tibia with LVI	**Neg** for AE1/1, CAM5.2, S100, Desmin, Actin, CD34 & CD31. SMA and Caldesmon—focal weak positive. **FISH** for SS18 Neg	Extracorporeal irradiation of the tibia, prior to excision	Development of distant metastasis plus 3 recurrent episodes; last FU in 2015	Solid epithelioid and pleomorphic	No
	MFS-38		Shin	Rec	Feb, 2014		**aCGH-** Gains in 3p, 4p, 6p, 7p, 8q, 11p, 11q & 13q; Ampl in 4q; Loss in 14q			Increase pleomorphism	No
Set 2	MFS-84	F60	Buttock	Primary	April, 2013	Myxofibrosarcoma, 85 mm, Grade 3	**Neg** for AE1/3, CAM5.2, EMA, S100, HMB45, Melan A, Actin, Desmin, CD34 &CD31. Ki-67- 40%	Nil prior to resection; enlarging mass presentig for 4 months prior to dx	In 2014 mets to pleura; last FU in 2014	With pleomorphic giant cells	No
	MFS-47		Frontal	Mets	March, 2014					Cellular and pleomorphic	No
Set 3	MFS-26	M65	Ulna	Rec 1	July, 2010	Recurrent myxofibrosarcoma, 20 mm, Grade 3	**Pos** for vimentin and focal desmin. **Neg** for Pan-CK, S100, Melan A, Actin, SMA, CD31 & CD34. Ki-67—hot-spots of 70%	No therapy prior to Rec 1	Primary MFS dx in 2006; After recurrence in 2012, no further Rec/Mets. FU in 2020 free of disease	Higly spindled and pleomorphic	Yes, mod
	MFS-74		Forearm	Rec 2	Feb, 2012			Amputation; prior radiotherapy and grafting		Spindled and a few pleom cells	No
Set 4	MFS-92	M65	Buttock	Primary	Jan, 2011	Myxofibrosarcoma, 115 mm, Gde 3; myoid differentiation	**Neg** for PanCK, S100, HMB45, Melan-A, CD34, CD31, Actin & Desmin. Pos for SMA focally	No prior therapy	Last FU in 2013	Epithelioid	No
	MFS-72		Thigh	Rec	Aug, 2012			Radio/chemotherapy & amputation		Similar histology	No
Set 3	SS1-B	F31	Femur	Primary	Dec, 2012	Synovial sarcoma, 150 mm	Focally **pos** for AE1/AE3 & EMA, BCL2 & CD99. **Neg** for CAM 5.2, CK19, S100, Melan A, Actin, Desmin, SMA, CD34 & CD31. **FISH** Pos SS18. **aCGH = **No gains, losses or amplifications	No prior therapy	Further lung mets in 2015 & 2017; developed chemotherapy-induced cardiomiopathy and heart failure and died in 2017	Monophasic, spindled cellular	No
	SS1-C		Lung	Mets 1	Oct, 2013			Chemotherapy		Similar histology	No
	SS1-D		Lung	Mets 2	Oct, 2013		aCGH = No gains, losses or amplifications			Similar histology	No
	SS1-E		Thigh	Rec	Aug, 2014					Similar histology	No
	SS1-G		Lung	Mets 3	March, 2015					Similar histology	No
Set 1	RMS-17	M17	Para- testicular	Primary	March, 2014	Embryonal rhabdomyosarcoma, 65 mm,	**Pos** for Desmin, Myogenin, focal MyoD1 & Actin. **Neg** for SMA, S100, PanCK, Calretinin, CD34, CD31, OCT3/4, PLAP, LCA, CD30 & ALK-1. **FISH** for FOXO1 neg	No prior therapy		Pleomoprhic epithelioid, w giant cells	No
	RMS-18		Pelvic LN	Mets	April, 2014			Chemo-radiotherapy after surgery	No further FU (2014)	Similar histology	No
Set 2	RMS-01	M57	Spermatic cord	Primary	Jan, 2016	Pleomoprhic rhabdomyosarcoma, 30 mm	**Pos** for Desmin, Myogenin & MYO-D1; focal SMA and S100. **Neg** for HMB45, Melan-A, PanCK, EMA, ERG, SOX10 & SALL4. INI1 preserved	No prior therapy	Mets to lungs; abdominal LN disection negative	Highly pleomorphic, epithelioid	Yes, high
	RMS-08		Brain	Mets	June, 2018					Similar histology	No

Mets: Metastasis; Gde: Grade (FNCLCC system); Pos: Positive; Neg: Negative; SMA: Smooth Muscle Actin; PR; Progesterone Receptor, ER: Estrogen Receptor; TILs: Tumour-infiltrating lymphocytes; Dx: diagnosis; FU: Follow-up; aCGH: array comparative genomic hybridization; Amp: Amplification; FISH: Fluorescent in-situ hybridization (break-apart probes).

### FFPE DNA extraction, bisulphite conversion and restoration

For DNA extraction, 1 mm tissue cores (n = 6–10 per case) were taken from FFPE tumour blocks using a tissue microarrayer (TMA) on samples with at least 50% of tumour cellularity. Specific details of DNA extraction, bisulphite conversion and restoration can be found in supplementary methods. Briefly, DNA was extracted using the ReliaPrep FFPE gDNA Miniprep System (Promega, Madison, WI) and DNA integrity was assessed by real time PCR (Infinium FFPE QC Kit, Illumina, San Diego, CA). Five-hundred ng of DNA was bisulphite-converted using the EZ DNA Methylation kit (Zymo, Irvine, CA). The effectiveness of bisulphite conversion was assessed by methylation specific PCR following the method of Esteller et al*.*^28^. Infinium HD FFPE Restore kit (Illumina, San Diego, CA) was used for DNA restoration.

### Methylation array and bioinformatics

Genome-wide DNA methylation profiling was performed on restored DNA using the Illumina Infinium MethylationEPIC BeadChip (Illumina, Inc., San Diego, CA, USA), which interrogates over 850,000 methylation sites, including CpG islands. The BeadChips were scanned using an Illumina iScan and analyses were undertaken using the R statistical environment (v. 3.5.1)^29^ and the ‘lumi’ Bioconductor package^30^. As tissue replicates were not feasible for this disease model we were restricted to direct comparisons of the tumour sites. To investigate the distribution of CpG sites, a beta-value difference of > 0.2 was selected to define probes with differential methylation between groups analysed as previously described^16,20^. Beta-values range from 0 to 1, where 0 indicates unmethylated and 1 indicates fully methylated^31^. The DMRforPairs^32^ package in R was used to identify differentially methylated regions (DMRs) of the genome. The DMRforPairs model is specifically designed to assess samples where replicates are not available. ‘Regions’ were defined as those with a minimum of 4 probes within a < 200 bp distance of each other with a median difference in M values > 1.4 between the samples of each Set. Regions were considered significant when the Benjamini Hochberg corrected *p* values were < 0.05. DMRs were sorted by adjusted *P* value and the top most significant region was provided. Variance analysis was used to rank probes by the greatest absolute difference and was calculated on the matrix of M-values in each pair of samples using the var() command in R. The top 50 and 500 most variable probes with the largest difference in methylation were extracted from each sample and displayed on a heatmap (using M-Values) to view the methylation patterns among the top contributors. Functional analysis using the KEGG Pathway database for pathway mapping^33,34^ was used to identify the top 500 most variable probes in each set. Please refer to Suppl. Methods for full details.

### Copy number variation (CNV)

This was performed by Array chromosomal genomic hybridization (aCGH) on 5 samples, but only 2 matched samples (SS) using SurePrint G3 Human CGH Microarrays, 8 × 60 K (Agilent, Australia) and CytoGenomics Software (Agilent, Australia) for analysis (Suppl. Methods). For all additional samples the program Conumee in R (http://bioconductor.org/packages /release /bioc /html/conumee.html) was used to obtain CNV. Controls were taken from a publicly available EPIC submission of GIB (NA12878; ID:GSE103498) consisting 4 replicates. The CNV calling algorithm underlying Conumee -DNACopy- uses a threshold of < -0.3 and > 0.3 for copy loss and gain, respectively.

## Results

### Clinical pathological samples

All LMS cases (n = 7 patients; n = 15 samples) presented in females with age ranging from 32 to 62 and primary site including uterus (n = 5), omentum (n = 1; set1) and kidney (n = 1; set7). Metastatic disease presented within 1 to 5 years of the initial diagnosis to the lung (n = 4), abdominal cavity (n = 2) or vagina (n = 1; Table 1). Five sets were comprised of primary tumour and matched metastatic deposit whilst two additional cases were comprised of metachronous metastatic deposits (set 6: Mets1 & Mets2; set 5: Mets1, Mets2 & Mets3). None of the primary tumours received therapy prior to resection. All metastatic cases had received chemo and/or radiotherapy (Table 1). Histopathological assessment of paired samples revealed that no significant TILs (> 10%) were present in any of the samples and that morphological differences across the matched samples were present in 5 of 7 cases (except for sets1 & 6; Supp. Figure 1 and Table 1).

**Figure 1:**
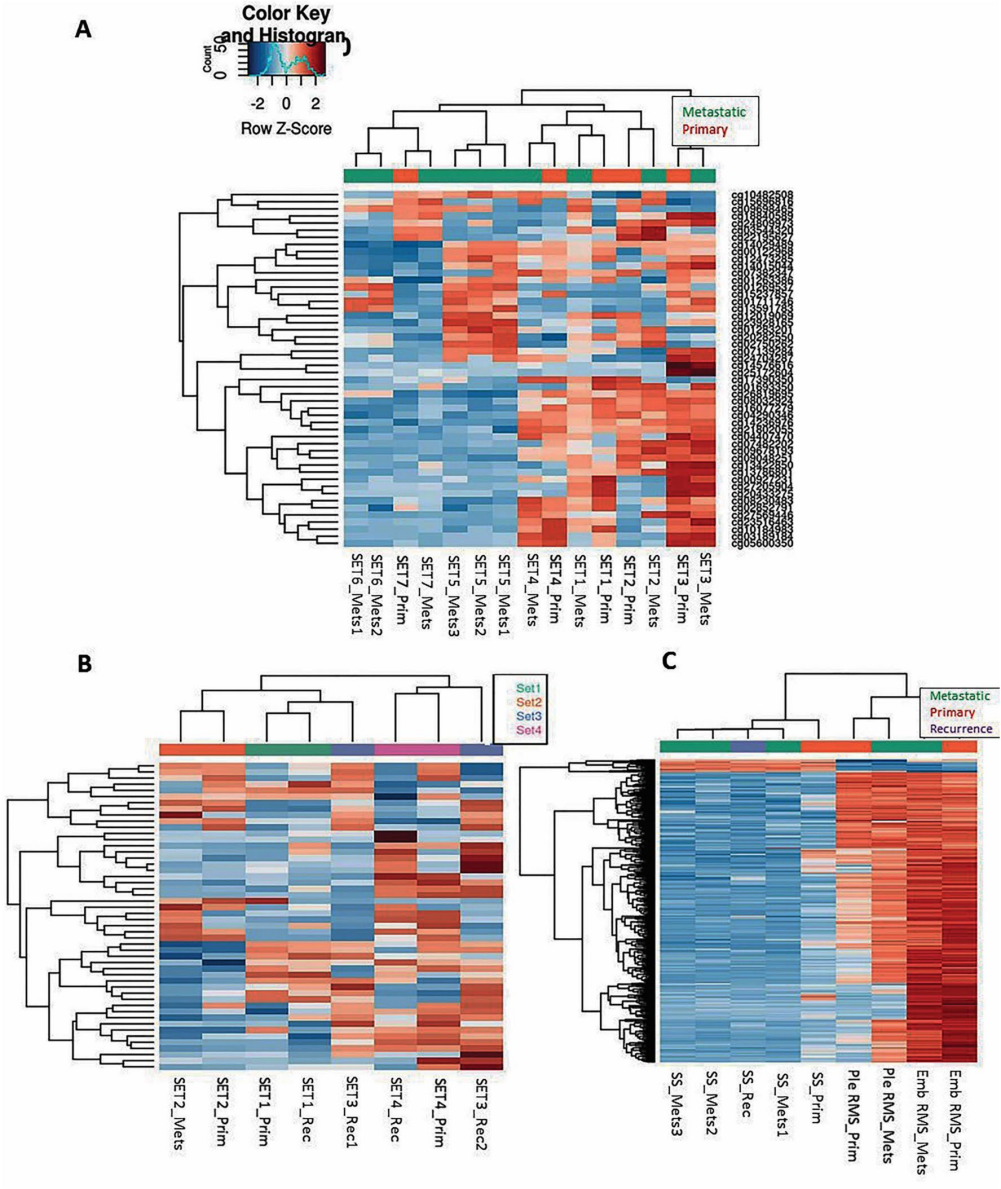
Heatmaps of the top 50 most variable probes across the samples using hierarchical clustering to visualise sample similarity and clustered probes generated according to tumour type: Leiomyosarcomas (**A**), Myxofibrosarcomas (**B**) and synovial sarcoma (SS) and Embryonal (Emb) and Pleomorphic (Ple) rhabdomyosarcomas (RMS; **C**). Red and blue represents hypermethylation and hypomethylation, respectively. Clustering heat maps created using M values. Illumina Infinium MethylationEPIC BeadChip and R statistical environment (v.3.5.1).

**Table 3:** Summary of methylation levels, DMR of Pairs and Variance Analysis in all paired comparisons analysed.

Sample Set	Methylation levels		DMRforPairs: chromosomal regions											
Set	Z score	Cor (r =)	Chrom; bp size region	Genes in DMRs	P value	B median—*Primary	B median Mets/Rec	M med Primary	M med Met/Rec	Mets/rec versus primary	Gene name	Relation to island & Reg Feature Group	UCSC RefGene Group	CNV (seg mean)
LMS_Set1	9938	0.878	Chr 6 (897 bp)	HLA-J	*P* = 4.13 × 10^–6^	0.127	0.344	− 2.787	− 0.933	Hypermethylated	Major Histocompatibility Complex, Class I, J	S shore / island	Body	Prim: 0.159 / Mets: 0.186
LMS_Set2	29,418	0.825	Chr 22 (722 bp)	DGRC6 & PRODH	*P* = 1.03 × 10^–4^	0.094	0.336	− 3.273	− 0.985	Hypermethylated	Proline Dehydrogenase	island; promoter- assoc	TSS1500, TSS200/ 5′UTR;1stExon	**Prim: − 0.032 / Mets: 0.339**
LMS_Set3	19,332	0.844	Chr 13 (614 bp)	PCDH20	*P* = 3.97 × 10^–4^	0.076	0.342	− 3.608	− 0.946	Hypermethylated	Protocadherin 20	OpenSea	TSS1500, TSS200/ 5′UTR;1stExon	Prim: 0.24 / Mets: 0.17
LMS_Set4	17,152	0.851	Chr 14 (761 bp)	HSPA2 & PPP1R36	*P* = 1.55 × 10^–3^	0.187	0.492	− 2.123	− 0.047	Hypermethylated	Heat Shock Protein Family & Protein Phosphatase 1 Regulatory Subunit 36	island, N-Shore; promoter-assoc	TSS1500, TSS200/ 5′UTR;1stExon	Prim: − 0.001 / Mets: 0.015
LMS_Set5a (M2 vs. M3)	7840	0.877	Chr 6 (715 bp)	FAM50B	*P* = 1.28 × 10^–3^	0.28 (M2)	0.714 (M3)	− 1.355	1.321	Hypermethylated (M3 vs. M2)	Family With Sequence Similarity 50 Member B	island, N-Shore; promoter-Assoc	TSS1500, TSS200	Mets2: 0.066 / Mets3: 0.101
LMS_Setb (M1 vs. M2)	7840	0.889	Chr 6 (400 bp)	DAXX	*P* = 2.25 × 10^–3^	0.523 (M1)	0.191 (M2)	− 2.084	0.134	Hypomethylated (M2 vs. M1)	Death Domain Associated Protein	island, N-Shore	Body	Mets1: 0.133 / Mets2: 0.066
LMS_Set6	1489	0.953	Chr 14 (523 bp)	LTB4R & CIDEB	*P* = 5.31 × 10^–3^	0.289 (M1)	0.087 (M2)	− 1.292	− 3.392	Hypomethylated (M2 vs. M1)	Leukotriene B4 Receptor & Cell Death Inducing DFFA Like EffectorB	island; promoter -assoc	5′UTR;Body;1stExon; TSS1500;TSS200	**Mets1: 0.458 / Mets2: − 0.043**
LMS_Set7	5480	0.9	Chr 3 (429 bp)	BTD & HACL1	*P* = 1.39 × 10^–4^	0.106	0.055	− 3.071	− 4.09	Hypomethylated	Biotinidase deficiency & 2-Hydroxyacyl-CoA Lyase 1	island; promoter -assoc	5′UTR;TSS200;1stExon; TSS1500	Prim: 0.17 / Mets: 0.2
MFS_Set1	16,538	0.81	Chr 7 (937 bp)	MEST	*P* = 9.45 × 10^–4^	0.649	0.368	0.885	− 0.779	Hypomethylated	Mesoderm specific transferarse	island	TSS1500;5′UTR; 1stExon	**Prim: 0.254 / Rec: 0.321**
MFS_Set2	24,993	0.845	Chr 14 (295 bp)	miR-379–656	*P* = 3.49 × 10^–2^	0.67	0.23	1.02	− 1.743	Hypomethylated	MicroRNA cluster (C14MC) / miR-379/miR-656	OpenSea	TSS200;TSS1500	**Prim: 0.356 / Mets: 0.262**
MFS_Set3	29,919	0.74	Chr 2 (675 bp)	SNRPG & FAM136A	*P* = 9.31 × 10^–4^	0.061 (Rec 1)	0.115 (Rec 2)	− 3.954	− 2.946	Hypermethylated (Rec2)	Small Nuclear Ribonucleoprotein Polypeptide G & Family With Sequence Similarity 136 Member A	S-Shore, N-shore; promoter-assoc	1stExon;5′UTR, TSS200, body	Rec1: 0.043 / Rec2: 0.234
MFS_Set4	21,246	0.841	Chr 12 (884 bp)	CCND2	*P* = 2.02 × 10^–4^	0.074	0.311	− 3.654	− 1.147	Hypermethylated	Cyclin D2	island	TSS1500	Prim: 0.145 / Rec: 1.015
SS (Prim vs. M1)	8138	0.91	Chr 11 (991 bp)	WT1-AS	1.79 × 10^–2^	0.203 (Prim)	0.068 (Mets)	− 1.972	− 3.776	Hypomethylated	WT1 antisense RNA	S-Shore, N-shore, island	Body	Prim: 0.097 / M1:− 0.198
SS (Prim vs. M2)	6742	0.913	Chr 6 (369 bp)	TNXB & SP2D6	8.74 × 10^–3^	0.216 (Prim)	0.076 (Mets)	− 1.86	− 3.621	Hypomethylated	Tenascin XB	island, S-Shore	Body	Prim: 0.263 / M2:0.061
SS (Prim vs. M4)	8177	0.908	Chr 12 (499 bp)	RP11-366l20.2	2.05 × 10^–2^	0.227 (Prim)	0.056 (Mets)	− 1.77	− 4.086	Hypomethylated	N/A	OpenSea	TSS1500;TSS200;Body, 5′UTR	Prim: 0.126 / Mets4: − 0.176
SS (Prim vs. Rec)	6719	0.917	Chr 15 (518 bp)	DUOX11 & DUOXA1	*P* = 7.48 × 10^–3^	0.171 (Prim)	0.051 (Rec)	− 2.275	− 4.231	Hypomethylated	Dual Oxidase 11 & Dual Oxidase maturation Factor 1	island	TSS200, TSS1500; 5′UTR, 1stExon	Prim: 0.054 / Rec: 0.061
Emb-RMS	7024	0.915	Chr 20 (165 bp)	No genes	*P* = 1.92 × 10^–2^	0.144	0.051	− 2.573	− 4.223	Hypomethylated	N/A	Island; promoter- assoc	TSS200, TSS1500	Prim: − 0.161 / Mets: − 0.102
Ple-RMS	29,429	0.839	Chr 10 (426 bp)	UTF1, VENTX & MIR202HG	*P* = 1.08 × 10^–4^)	0.157	0.469	− 2.441	− 0.181	Hypermethylated	VENT Homeobox, Undifferentiated Embryonic Cell Transcription Factor 1 & MIR202 host gene	Island	TSS1500	**Prim: 0.115 / Mets: − 0.369**

P: Primary; M: Metastasis; LMS: Leiomyosarcoma; MFS: Myxofibrosarcoma; SS: Synovial sarcoma; RMS: Rhabdomyosarcoma; Emb: Embryonal; Ple: Pleomorphic; Cor: Correlation; bp: base pair; Chr: chromosome. DMRs: Differentially methylated regions; Mets: Metastasis; Rec: Recurrence; Relation to Island: The location of the CpG relative to the CpG island. Reg (Regulatory) Feature Group: Description of the regulatory feature as provided by the Methylation Consortium. Shore = 0–2 kb from island; Shelf = 2–4 kb from island; N = upstream (5′) of CpG island; S = downstream (3′) of CpG island. UCSC Ref Gene Group: Gene region feature category describing the CpG position, from UCSC. When more than one feature is listed indicates different target gene transcripts. 5′UTR = Within the 5′ untranslated region, between the TSS and the ATG start site; Body = Between the ATG and stop codon; irrespective of the presence of introns, exons, TSS, or promoters; TSS200 = 0–200 bases upstream of the transcriptional start site (TSS); TSS1500 = 200–1500 bases upstream of the TSS. 3′UTR = Between the stop codon and poly A signal (As per descriptions of the Infinium MethylationEPIC Array manifest file column headings).

MFSs (n = 4 patients; 8 samples) presented equally in males and females, age ranging 60 to 76 years and all were FNCLCC grade 3 with Rec/Met deposits presenting 1 to 2 years apart. Apart from one case with brain metastasis (set 2) the remaining 3 sets comprised recurrent tumours at the same anatomic site (Table 2). Three of four cases did not receive therapy prior to the resection with the remaining patient undergoing irradiation prior to resection (Table 2). On histopathological assessment 3 of 4 cases (except for set 4) demonstrated differences across paired samples (Supp. Figure 2). TILs were only seen in one case (set 3 in Rec1 but not in Rec2).

**Figure 2:**
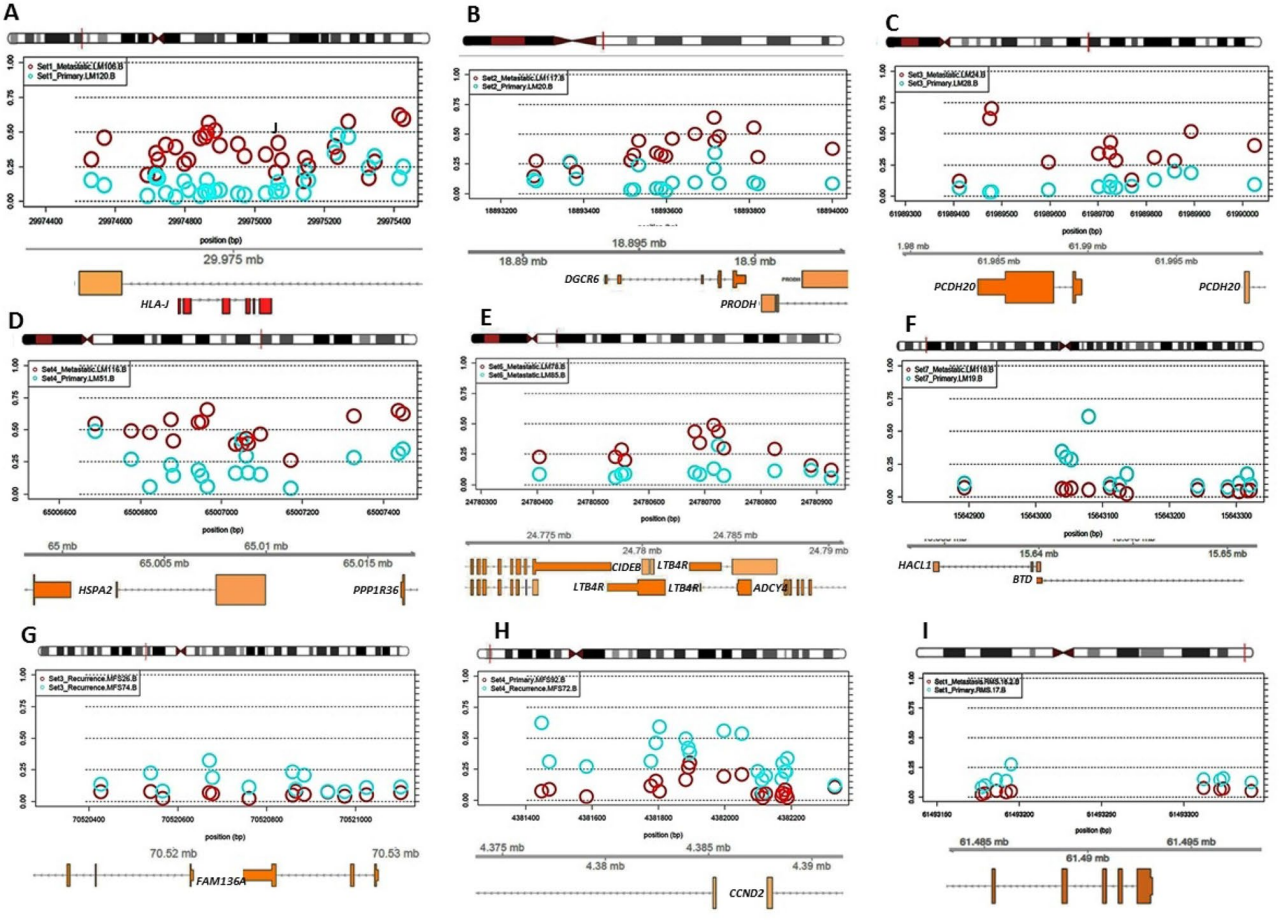
Beta values, probe distribution and genomic annotation for significant differentially methylated regions (DMRs) of selected paired samples. Leiomyosarcomas (LMS) sets 1 (**A**: *HLA-J* in Chr 6 [897 bp region]), 2(**B**: *DGRC6* & *PRODH* in Chr 22 [722 bp region]), 3(**C**: *PCDH20* in Chr 13 [614 bp region]), 4(**D**: *HSPA2* & *PPP1R36* in Chr 14 [761 bp region]), 6(**E**: LTB4R & CIDEB in Chr 14 [523 bp region]) & 7(**F**: BTD & HACL1 in Chr 3 [429 bp region]). Beta values are plotted in blue (primary) and red (Metastatic: Mets). Set 6 (**E**) includes paired Mets (Mets1: red; Mets2: blue). Myxofibrosarcoma (MFS) sets 3 (**G**: *SNRPG* & *FAM136A* in Chr 2 [675 bp region]) & 4(**H**: *CCND2* in Chr 12 [884 bp region]). Sets 4 (primary red; Recurrence [Rec] blue); & set 3 (Rec1 red; Rec2 blue). Embryonal rhabdomyosarcoma (**I**: Chr 20, 165 bp region; primary blue; Mets red).

Both cases of rhabdomyosarcomas (n = 2 patients; 4 samples) originated in the spermatic cord of 17-(Emb-RMS) and 57-year old (Ple-RMS) males who did not receive therapy prior to surgery. Metastasis developed in the para-aortic lymph nodes (1 month: Emb-RMS) and brain (2 years: Ple-RMS; Table 2). Histopathological assessment revealed no histomorphological differences across paired samples (Supp. Figure 3). However, Ple-RMS was noted to have dense TILs in the primary tumour not seen in the brain metastasis.

**Figure 3:**
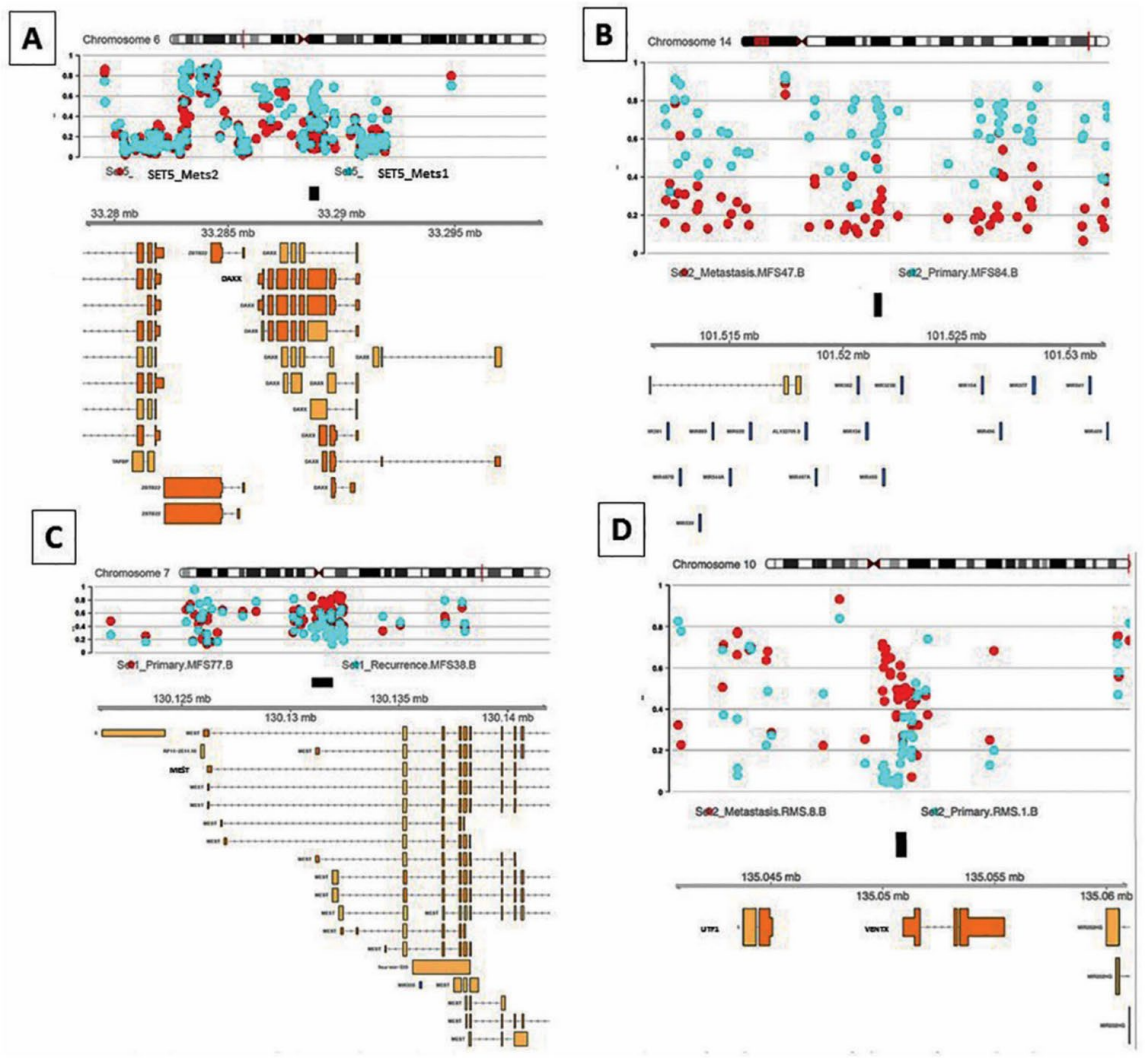
B-values and genomic annotation for some of the top differentially methylated regions (DMRs) of the genome detected in Leiomyosarcoma (LMS), myxofibrosarcoma (MFS) and Pleomorphic Rhabddomyosarcoma (Ple RMS). In LMS Set 5 (**A**) the top result was in the *DAXX* gene on chromosome (chr) 6. In MFS top DMRs were located on chr. 14 within the human microRNA cluster, *C14MC* (Set2: **B**) and chr. 7 containing the Mesoderm specific transferase (*MEST*) gene (Set1: **C**). In Ple-RMS (**D**), the top DMR was located to chr.10 around the *UTF1, VENTX* and *MIR202HG* genes.

One case of synovial sarcoma (n = 5 samples used for the study) presented on the thigh of a 31 year-old woman, who developed multiple metachronous lung metastases (> 10 metastatic deposits resected on 6 separate operations) including recurrence within a 3 year period. No therapy was received prior to the resection of the primary tumour. She received chemotherapy and developed chemotherapy-induced cardiomyopathy and heart failure, and died 3 years after the primary diagnosis. No significant morphological differences, including complete absence of TILs, were identified across the different stages of progression (Table 2 and Supp. Figure 4).

**Figure 4:**
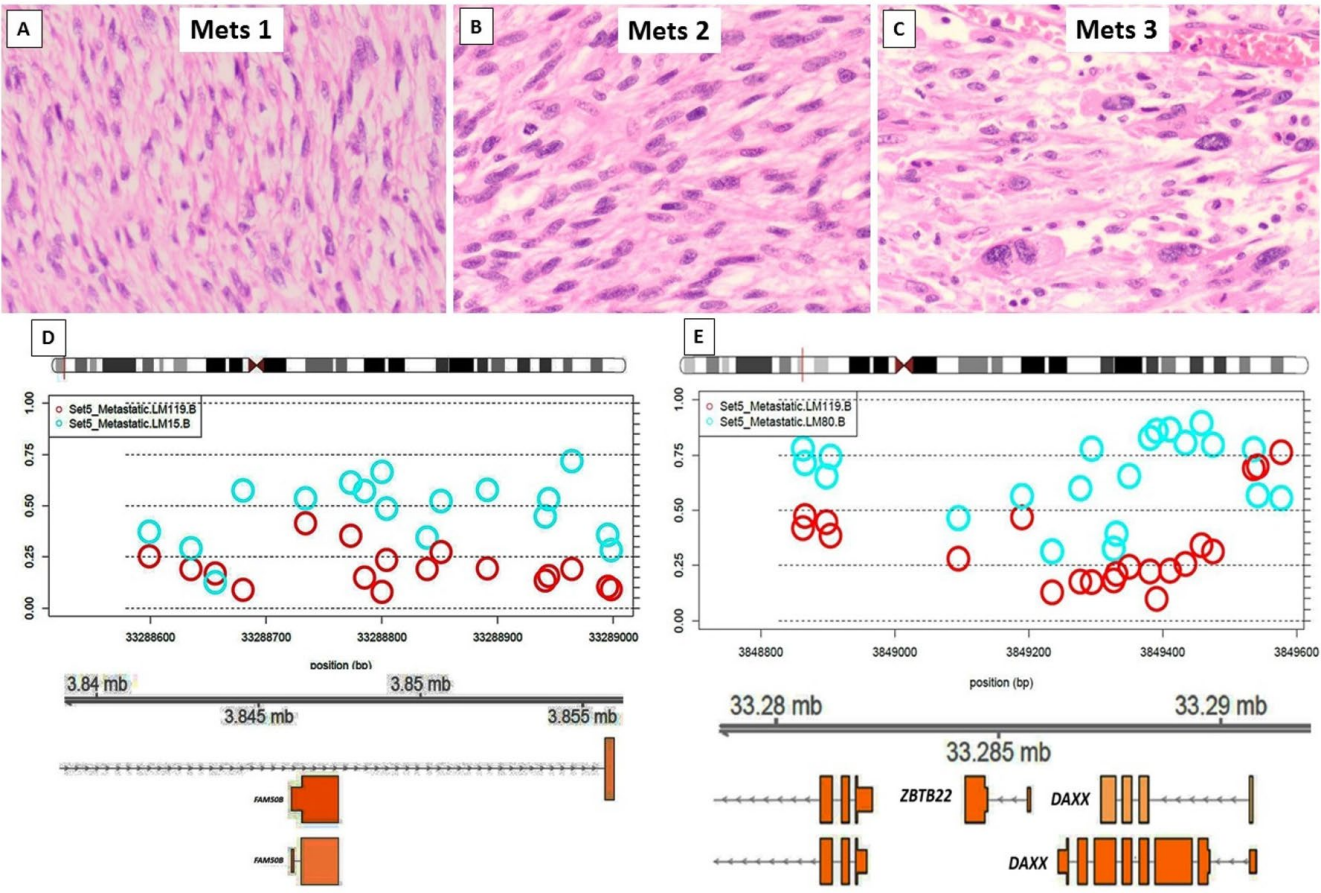
Metastatic leiomyosarcoma (Set5) from a primary uterine with all 3 metastatic deposits (Mets 1–3) occurring in the abdominal cavity (peritoneum and omentum), each at 2 years interval. Histological progression with Mets 1 (**A**) showing a low-grade spindle cell tumour, which increased cellularity and hyperchromasia in Mets2 (**B**) and high grade pleomorphic giant cells in Mets3 (**C**). B-values, probe distribution and genomic annotation of differentially methylated regions (DMRs) across metastases (**D**–**E**). Mets1 versus Mets2 (**D**) shows DMR on chr 6 containing *DAXX*, which showed hypomethylation in Mets2 (red) versus Mets1 (blue; **D**). A separate hypermethylated region on the same chromosome was identified in Mets3 (blue) versus Mets2 (red) containing *FAM50B* (**E**).

### Global methylation variation and histopathological correlation

Successful methylation profiles were obtained from all 32 samples. (Fig. 1 and Supp. Figure 5). Due to the reduced sample quality of FFPE tissue, between 72 and 88% of the Epic array probes remained after filtering and normalization across samples (LMS set: 655,934; MFS set: 625,421 and SS/RMS set: 760,009).

**Figure 5:**
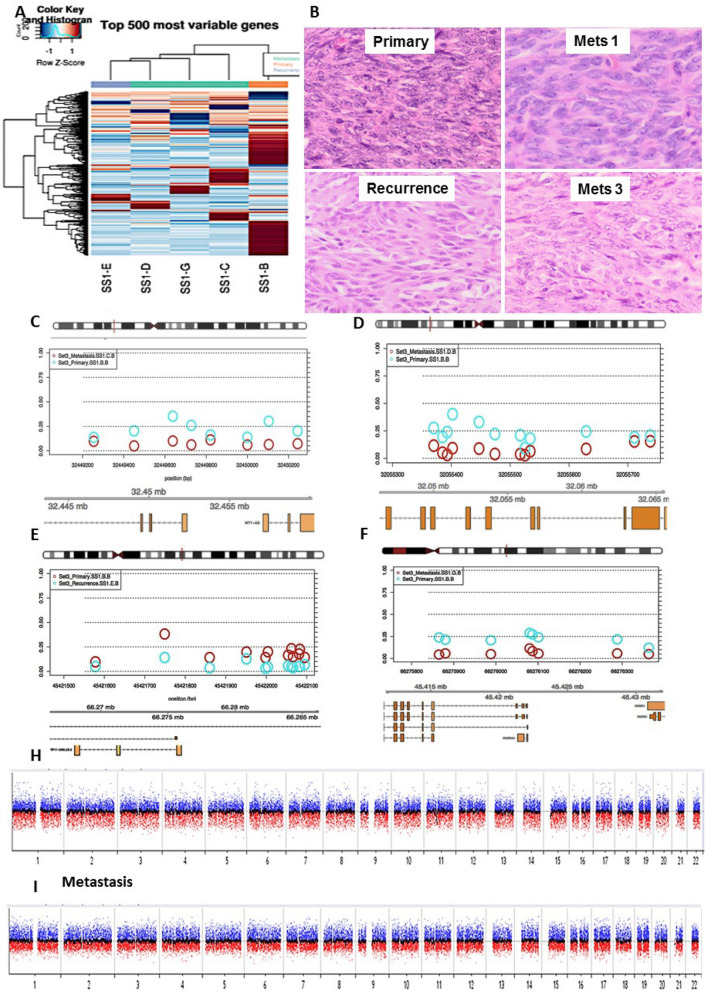
Heatmap of the 500 most differentially methylated probes (**A**) between primary synovial sarcoma (SS; SS1-B), recurrence (SS1-E), Mets1 (SS1-C), Mets2 (SS1-D) & Mets3 (SS1-G; M-values). Illumina Infinium MethylationEPIC BeadChip and R statistical environment (v.3.5.1). Histological images of 4 of 5 of the samples used for methylation array (**B**). B-values, probe distribution and genomic annotation of differentially methylated regions (DMRs: **C**–**F**). Primary versus Mets1 containing *WT1-AS* in Chr 11 (991 bp region: **C**). Primary versus Mets2 contatining *TNXB* & *SP2D6* in Chr 6 (369 bp regionL: **D**); Primary versus Rec containing *DUOX11* & *DUOXA1* in Chr 15 (518 bp region: **E**); Primary versus Mets3 contatining *RP11-366l20.2* in Chr 12 (499 bp region: **F**). Primary beta values are plotted in blue and Metasatic/Recurrence beta values are plotted in red. Array comparative genomic hybridisation plots of primary SS (**G**) and Mets2 (**H**), demonstrating no significant chromosomal gains, losses or amplifications in any of these samples. Genome View, numbers correspond to chromosomes 1–22, middle black line indicates the average log ratio for the probes (SurePrint G3 Human CGH Microarrays 8 × 60 K and CytoGenomics Software (Agilent, Australia).

Variation in global methylation levels (defined by a Z-score r =  < 0.9; Table 3) were identified across almost all matched samples of LMSs (n = 5/7), all MFSs (n = 4/4) and Ple-RMS. MFSs showed the largest global methylation variance (Z-scores ranging from 0.74 to 0.84) followed to similar degree by Ple-RMS and 5 of 7 LMSs (Z scores between 0.82 and 0.88). Matched samples from SS and Emb-RMS showed r =  > 0.9, indicative of a more similar methylation profile between the primary tumour and their corresponding Mets/Rec deposit.

Histopathological review on H&E-stained histological sections (Suppl. Figures 1–4) of the paired Met/Rec deposits for each individual set demonstrated correlation between higher variation in global methylation (Z-score < 0.9) and the acquisition of morphological differences in subsequent Met/Rec tumours in 5/7 LMSs & 3/4 MFSs. Concordantly, lower variation in global methylation (Z-score > 0.9) coincided with lack of histomorphological changes in Rec/Met disease for both RMSs, SS & 1 LMS [set6]. This trend was absent in only 2 of 14 of the sets, which showed either marked morphological variation without significant variation in global methylation (LMS set 7; r = 0.9) or absence of histological differences but marked variation in global methylation (MFS set 4; r = 0.84). This analysis indicates a level of correlation between variation in methylation levels and morphological changes identified by light microscopy on Rec/Met tumours during progressive disease in 85.7% of all samples (*P* =  < 0.01; Fisher’s exact test).

A heatmap of the top 50 and 500 most variable probes according to tumour type demonstrated that, as expected, matched samples (sets) clustered together indicating that these were more closely correlated with each other than to stages of progression (i.e. primary vs. Mets; Fig. 1 and Supp. Figures 5–6). Two of the paired MFS samples clustered in a more dispersed fashion than expected for matched samples, confirming marked differences in methylation levels across Mets/Rec disease.

### Differentially methylated regions and methylation patterns across paired Met/Rec samples for individual sarcoma types

#### Leiomyosarcomas

The following differentially methylated regions (DMRs) and probes within these regions were differentially methylated between primary and metastatic LMSs: *BTD* and *HACL1* on chromosome (chr) 3; *HLA-J, FAM50B* and *DAXX* across two different DMRs on chr 6; *PCDH20* on chr 13; *HSPA2, PPP1R36, LTB4R* and *CIDEB* across two different regions on chr 14; and *DGRC6* and *PRODH* on chr 22. Although specific DMRs varied across the sets, consistently increased methylation levels were identified in Mets versus primary tumours for most of the identified DMRs (Table 3 and Figs. 2, 3). Four of the five primary LMS analysed were essentially unmethylated (B values ranging from 0.07 to 0.18) whilst the paired metastases demonstrated a gain in methylation levels (B values ranging from 0.33 to 0.49). Only one primary LMS displayed minimal change in the methylation levels for the DMR identified (B value of 0.1 in the primary vs. 0.05 in metastasis) but coincidently, this case did not harbour variance in global methylation (set7: renal LMS). Concordantly, the additional cases with only sequential metastatic deposits available for comparison (Sets 5&6) demonstrated elevated baseline methylation levels in Mets1 (B values of 0.28 and 0.52, respectively) similar to all metastatic LMS samples with a consistent trend of hypermethylation in all Mets1 deposits when compared to primary LMSs. However, in subsequent metastatic disease (Mets2), methylation decreased to lower levels for the specific DMRs identified (which included probes for *DAXX*, *LTB4R* & *CIDEB*) in both cases (Table 3). Set5, which had a further metastatic deposit (Mets3) occurring 2 years apart, showed a further hypermethylated region including *FAM50B*, which developed in Mets3 but which was not identified in Mets1. This is indicative of the late acquisition of a differentially methylated clone (Fig. 4).

Variance analysis (Suppl Table 1) revealed that *PTPRN2, KCNQ1* and *MCF2L,* all located to CpG islands, were the most commonly shared probes over all paired comparisons.

We then correlated DMRs identified with underlying CNVs for the specific chromosomal regions to determine whether CNVs would account for increased or decreased methylation across paired samples (Table 3 and Suppl. Figures 8–11). Apart from 2 samples (set2 with *DGRC6* & *PRODH* at Chr 22 & set6 with *LTB4R* & *CIDEB* at Chr 14), all remaining samples did not show CNVs in the identified chromosomal regions. This excludes chromosomal aberrations as the underlying mechanism of differentially methylated genes in 5 of 7 LMS pairs (71% of the pairs and in 11 of 15 individual samples [73%]). For set 2, hypermethylation of DMR in the metastatic deposit correlated with copy gain within the same chromosomal region and concordantly for set 6, copy loss in Mets 2 correlated with hypomethylation of the specific chromosome. However, CNV in both cases was only above the call threshold (Table 3) and not indicative of amplification.

Finally, enriched KEGG pathway analysis identified hypermethylated and hypomethylated pathways (Suppl. Tables 2–3), but which were not specific neither to the LMS group nor sarcomas in general. Moreover, although some of the pathways overlap in some of the sets, most of the top 10 pathways were unique to individual sets and not shared across most samples.

#### Myxofibrosarcomas

A highly variable number of DMRs were identified in Myxofibrosarcomas (Figs. 2, 3), which were not shared across the sets and included the following genes: *MEST* (chr 7), *C14MC* microRNA cluster (also known as miR-379–656; chr 14), *SNRPG and FAM136A* (both in chr 2) and *CCND2* (chr 12). The most variable probes identified through variance analysis were *TBX15* (chr1), *PLEC*1 (chr8) and *CDH15* (Suppl. Table 1).

Of the 500 most variable probes, most were uniquely observed within each sample with variable number of overlapping genes across the sets but no common genes across all cases (Suppl. Figure 7). Whilst upregulated pathways included cytokine receptor and metabolism-related pathways, some of the downregulated pathways included cell adhesion (Suppl. Tables 2–3). Most of the identified pathways were unique to individual sets.

CNVs for the selected DMRs (Suppl. Figures 12–13) showed that 2 of 4 samples (set1 with *MEST* at Chr 7 & set2 with miR-379–656 at Chr 14) showed minor changes in CNVs across the pairs (Suppl. Figure 12). However, these changes were just borderline (just > 0.3 in both instances: Table 3) and their impact with regards to methylation levels is unlikely.

#### Rhabdomyosarcomas

In Ple-RMS, the top DMR was located on chr 10q including the *UTF1*, *VENTX* and *MIR202HG* genes (Figs. 2, 3; Table 3). This region was hypermethylated in the brain metastasis compared to primary Ple-RMS (B values: 0.46 vs. 0.15 in Mets). CNV analysis demonstrated copy loss in the metastasis (when compared to the primary tumour) in the same region as this DMR (Suppl. Figure 14). The most variable probe, through variance analysis, was located in the *NTRK3* gene (chr15). The most significant pathway identified by Reactome pathways analysis was the Receptor Tyrosine Kinase signalling followed by fibroblast growth factor family genes (*FGFR3* and *FGFR3c*) and WNT signalling. Additional KEGG pathway analysis is shown in Suppl. Tables 2 and 3.

In embryonal RMS, the top DMR was located on chr 20 including a 165 bp region, which showed decreased methylation in Mets compared to Primary (Beta value median: 0.14 vs. 0.05, respectively). No genes were identified in this region and no underlying copy number variation was identified (Suppl. Figure 14). Through variance analysis, the most variable probe was located in chr1 including the *OLFML2B* gene. KEGG pathway analysis is shown in Suppl. Tables 2 and 3.

#### Synovial sarcoma

The five samples analysed from this case (Primary, Mets1, Mets2, Rec and Mets3) were similar in their level of methylation but methylation changes and DMRs were also identified across stages of progression. Interestingly, Rec showed more similar methylation levels and clustered more closely to Mets than to the Primary tumour (Figs. 1, 5). *WT1-AS* (chr11 region) and *TNXB* and *SP2D6* (both within the same region on chr6) were in the top 10 differentially methylated genes between Primary versus Mets1, Mets2 and Rec. The primary sample revealed low methylation levels for both regions (B values 0.20–0.21), which markedly dropped in Mets1, Mets2 and Rec (0.06–0.07; Table 3). Two further differentially unmethylated regions were identified in Rec compared to Primary (Chr 15 including *DUOX11* and *DUOXA1*) and in Mets4 (Chr12 containing RP11-366l20.2). These regions were not detected in the remaining samples (Fig. 5). Through variance analysis, two additional probes were identified. *TBC1D5* was identified in primary versus Mets1-Mets3 but not in Rec whilst *CREB5* was identified in Rec only (Suppl. Table 1).

KEGG pathway analysis showed that enriched hypermethylated probes included insulin secretion and calcium signalling whilst pathways enriched with hypomethylated probes related to downregulation of cancer signalling pathways (Suppl. Tables 2–3).

Array CGH was performed on the primary SS and one of the metastatic deposits (Mets 2). This analysis confirmed, as expected, that SS is associated with simple karyotype as no significant chromosomal gains, losses or amplifications were detected neither in primary SS nor in Mets 2. Importantly, acquisition of complex genomic aberrations was not observed in Mets2 with the plots for both samples showing essentially identical appearance (Fig. 5). Thiese findings were also concordant with our CNV analysis using Conumee and the DNACopy calling algorithm (Table 3).

## Discussion

In this study we performed for the first time, genome-wide methylation comparative analysis of matched selected sarcoma samples obtained at different time points during the evolution of the tumours. Differential methylation was consistently identified in Met/Rec disease in all our matched samples (n = 14 pairs; 32 samples) reflecting the presence of objective epigenetic differences across primary and Met/Rec human tissue samples not previously reported.

Although it would have been ideal to perform complementary genomic analysis, no significant gene mutations and/or gene drivers were reported to occur in selected matched sarcoma samples in the only study published to date using whole-exome sequencing and SNP arrays (n = 20: myxoid liposarcoma, well differentiated liposarcoma and MFS). In that study, Hofvander et al.^24^, hypothesised that in the absence of genomic drivers, it is possible that methylation changes may play a major role in the metastatic cascade of sarcomas and our study aimed to address this hypothesis in spite of limitations due to FFPE-derived DNA and the small number of cases. Although sarcomas tend to develop late metastases, our cohort was enriched by sarcomas with aggressive behaviour as Met/Rec deposits occurred within 5 years after the initial presentation. We selected this biased cohort with the aim to identify key changes developing early during clonal evolution of aggressive sarcomas associated with recurrence and/or metastatic propensity.

Global methylation patterns varied according to sarcoma type. Almost all LMSs and Ple-RMS showed DMRs associated with low levels of methylation in the primary tumour (unmethylated) but with increased methylation in paired metastatic samples. Interestingly, low methylation has been identified in primary sarcomas of high-risk sarcoma patients with poor prognosis^12^ in line with our data. In the context of LMS, this was uniformly seen in all cases of initial metastatic disease (Mets1) but not maintained in subsequent metastatic deposits (Mets2 and Mets3) for the two cases analysed. The reverse trend was identified for SS and Emb-RMS, which revealed hypomethylation in Met/Rec disease compared to the primary tumour. Finally, no specific methylation pattern was identified for MFS paired samples, which showed the largest variation in methylation levels compared to any other sarcoma type and which was independent of the content of TILs and FNLCC grade as most tumours (7 of 8 samples) lacked TILs and were grade 3.

We identified differentially methylated regions and genes in LMSs, which were unrelated to underlying CNV indicative of primary epigenetic events for these specific samples. From our gene list, two genes have been previously linked to LMS sarcomagenesis—*DAXX* and *PTPRN2*. *DAXX* (Death Domain–Associated) forms a dimer with *ATRX* (Alpha-Thalassemia/Mental Retardation Syndrome X-linked) and dysfunction of this *ATRX/DAXX* complex is associated with alternative lengthening of telomeres (ALT), which occurs in a proportion of LMSs^35–37^. Moreover, loss of *ATRX* and/or *DAXX* has shown to predict aggressive clinical behaviour in LMSs and other smooth muscle tumours^36–38^. Accordingly, we identified hypomethylation of *DAXX* in Mets2 versus Mets1, which would correlate with gene loss / silencing in aggressive LMSs. *PTPRN2* (Receptor-type tyrosine-protein phosphatase N2), identified through variance analysis as one of the most differentially methylated probes shared across most LMS samples, has been previously linked to primary LMS^39^. In a single study in breast cancer cells, *PTPRN2* was shown to regulate actin dynamics, enhancing metastatic migration^40^ and based on this, we can hypothesise that *PTPRN2* is also likely to act as a key regulator in the metastatic cascade of leiomyosarcomas but functional analyses are required to confirm this. Methylation modifications in other of the identified genes (*PCDH20*, *CDEB*, *KCNQ1*, *MCF2L*, *LTB4R* and *PRODH*)^41–44^ have been described in carcinomas but their role in the pathogenesis of leiomyosarcoma progression has not been reported (apart from *PCDH2 and PRODH*^45^), and should be further explored. Finally, we also looked into overlapping genes in our list with other similar studies of primary LMSs. There are only 3 comparable studies using genome-wide methylation profiling^9,13,45^. The Cancer Genome Atlas Research Network, in their subset analysis on primary LMS^9^, identified that differences in methylation across these tumours were predominantly driven by anatomic location (uterine vs. soft tissue LMSs). Moreover, LMSs clustered in two groups with differential methylation signature, which resulted in differences in recurrence-free survival^9^. Although shared genes were not identified with our study, it is difficult to draw conclusions from this in view of the different study design and biological question. Miyata et al.^45^, on a methylation analysis of a limited number of normal myometrium, leiomyomas and leiomyosarcoma (n = 9 samples in total) identified global hypomethylation in LMSs, which is consistent with our findings, including overlapping genes (*PDE6B, PRODH, HSPA2, LTB4R, HEPACAM, RAP1GAP2, ADAM32, ADARB2, PDE9A, SPTBN1, VIPR2, KCNQ1, PTPRN2, MTMR7, MCF2L* and *KCNQ1*). Interestingly, hypermethylation of protocadherin genes was significant in their study^45^ and also identified in some of our samples (*PCDH20* and *PCDH*) but no previously identified in LMSs. We can hypothesise that some of these shared genes may be associated with progression or may represent key genes defining a malignant phenotype. Kommos et al.^13^, performed genome wide methylation for tumour classification in a range of uterine tumours including LMSs and carcinomas. Although specific genes were not discussed in their study (or in supplementary files) the authors showed a specific methylation signature in LMS, allowing classification according to histological type.

The most variable genes differed among each MFS pairs. The roles of *MEST*, *C14MC*, *FAM136A*, *SNRPG*, *CCND2*, *TBX15*, *PLEC1* and *CDH15* have not been previously reported in this sarcoma type. Gene silencing of *MEST*, a key regulator of IL-6/JAK/STAT3/Twist-1 pathway-mediated tumour metastasis, has been demonstrated in other sarcomas^46,47^ and concordantly, we observed hypomethylation for the DMR containing *MEST* in one of our sets. Downregulation of *C14MC,* also known as miR-379/miR-656 cluster, has been described in brain tumours^48^ and others^49^, and was significantly hypomethylated in the metastatic deposit in one of our cases. *SNRPG*, involved in mRNA splicing^50^, and other genes have been involved in carcinogenesis but methylation dysregulation in sarcomas has not been described before. As MFSs are known to be genetically unstable and characterised by the acquisition of multiple genomic and epigenomic aberrations during progression^10,11,51^, it is unlikely that individual genes and methylation modifications identified in these tumours are primary driving events. Our CNV analysis demonstrated low level of CNV for 2 of the 4 pairs but no evidence of gene amplification or homozygous deletions. Therefore, it is possible that copy gain/loss may account for some of the methylation differences in these samples but this is uncertain. Finally, we sought to identify common genes in our study with others published in primary MFS. Ogura et al.^10^, in the largest study of primary MFSs with epigenetic and genetic data (n = 41), identified 3 clusters with distinct methylation signatures. Although only one of their genes (*PLEC*) overlapped with ours, both our study and theirs, identified differential methylation of cell adhesion-related genes. Ogura et al.’s genes included *CCND1* and *CTNNB1* whilst genes from the same family in our cohort included *CCND2* and *CDH15* together with downregulation of cell adhesion pathways.

In our case of synovial sarcoma (n = 5 samples), differentially methylated regions and genes were identified between the primary tumour and Rec/Met disease (Fig. 5). *WT1-AS* and *TNXB* became unmethylated in Rec/Mets. *WT1-AS* is a long non-coding RNA and the antisense transcript of Wilms’ tumour 1 gene (*WT1*). Downregulation of this gene enables cell proliferation, migration, and invasion leading to cancer progression in multiple cancer types, which has been correlated with advanced disease and poor clinical outcome^52–56^. *TNXB* encodes a tenascin which promotes epithelial mesenchymal transition (EMT) and is downregulated in cancer^57,58^, wherein methylation correlates with gene expression^59–65^. In agreement with the known downregulation of these genes in cancer, both *TNXB* and *WT1-AS* were unmethylated in Mets/Rec samples of SS, indicative of gene loss/downregulation during progression of SS, which has not been previously identified in this tumour type. Importantly, *WT1-AS* can negatively regulate transforming growth factor β (*TGF-β*) and *TP53* and coincidently, *TNXB* also interacts with *TGF-β*, further facilitating EMT^57^. We can hypothesise that *WT1-AS* and *TNXB* epigenetic inactivation and downregulation in SS leads to activation of the *TGF-β* pathway. Accordingly, alterations in the TGF-β1/SMAD pathway and dysregulation of the EMT process, which enables cell invasion, migration, and proliferation, have been demonstrated in cell culture of SS cells^66^. Hence, TGF-β pathway inhibitors, already developed^67^, may potentially have effect in controlling metastatic/recurrent disease in SS. Further studies are required to confirm this. Additional genes (*DUOX11*, *DUOXA1* and *RP11-366l20.2*) were also identified as unmethylated in individual stages of progression (Rec and Mets3) but were not shared across the samples. This suggests that differentially methylated subclones are likely to arise due to epigenetic dysregulation during the evolution of SS. Finally, by performing aCGH on two of the samples (Primary and Mets2), we demonstrated that the development of differentially methylated regions in metastases occurred in the absence of the acquisition of unbalanced chromosomal abnormalities as Mets2 maintained a simple karyotype identical to that of the untreated primary tumour (Fig. 4).

We analysed two unrelated types of rhabdomyosarcoma and although methylation changes have been consistently proven in Emb-RMS, these have not been studied before in Ple-RMS. In Ple-RMS, differentially methylated genes between Primary and Mets included *VENTX, NTRK3* and *UTF1*. *UTF1* and *VENTX*, within a 10q region, showed hypermethylation in metastasis compared to the primary. Nonetheless, copy loss was also identified in this 10q region in the metastasis, which could potentially be the driving event leading to differential gene methylation. The homeobox protein *VENTX* is antagonist of the Wnt signalling pathway and has shown to be dysregulated in the immune cells of the tumour microenvironment (TME), being a potential target for immunotherapy (Reviewed in^68^). Interestingly, our case showed a high number of TILs in the primary tumour, which were completely absent in the brain metastasis. We can only hypothesise that *VENTX* downregulation may have played a role in altering the composition of the TME in this case and may be a potential therapeutic target in sarcomas associated with immune cell content. Although differences in the gene expression and methylation signatures of the TME are associated with significant prognostic differences in patients with STSs^12^, such differences in the TME are unlikely to explain the results in our remainder samples, as most of our cases (10 of 14 paired cases) were over-represented by so-called ‘cold tumours’, devoid of TILs. On the other hand, the role of *UTF1* (undifferentiated embryonic cell transcription factor 1), which plays a role in stem-cell development^69^ and carcinogenesis^70–72^, and *NTRK3* in RMS has not been described. *NTRK3* methylation has been reported in other tumours^73^ and although tyrosine kinase inhibitors are widely available for *NTRK*-translocated tumours, the effect of these TRK inhibitors has not been explored in tumours with *NTRK* gain or loss due to methylation. In Emb-RMS, *OLFML2B*, Olfactomedin-like 2B, was the most variably methylated probe. This has not been previously investigated in this tumour type but it has been described as an oncogene in gastric carcinoma^74^.

Our findings also identified a level of correlation between variation in methylation levels and ‘histomorphological progression’ across matched pairs. Specifically, we identified that acquisition of morphological features (increased pleomorphism, cellularity, nuclear hyperchromasia and/or change in cell morphology) in Mets/Rec correlated with higher degree of global methylation. Concordantly, lack of morphological changes during stages of progression (SS, Emb-RMS and LMS set6) correlated with lower variance in global methylation. As individual histomorphological features cannot be objectively quantitated, this conclusion is only observational.

Our study has significant limitations, which include the low number of cases for each sarcoma type and the history of chemo/radiotherapy, mostly for treatment of subsequent metastatic/recurrent tumours (Tables 1, 2). It is possible that the identified DNA methylation changes in our series have been treatment-induced but regardless of the initiating mechanism associated with the development of subclonal selection, it is possible that such changes may reflect common pathways followed by aggressive sarcomas prone to metastasise and/or recur. It is well recognised that ionizing irradiation acts as an epigenotoxic agent because it can induce the following: global DNA hypomethylation (which contributes to genomic instability); loci of hypermethylation in tumour-suppressor genes leading to repressed gene expression; deamination of methylated CpG islands resulting in development of point mutations; decrease in DNA methyltransferases; and generation of reactive oxygen species (Reviewed in^75–77^). Nonetheless, most of the current understanding on the effects of irradiation on DNA methylation derives from in-vitro and in-vivo experimental systems with no significant data addressing the translational relevance in human tissue samples. Although we performed detailed morphological characterisation of the samples, currently there are no specific histological markers detected on the cancer/sarcoma cells, which allows distinction between changes secondary to treatment effect versus dedifferentiation or histological transformation/progression. With regards to the low number of samples included in our series, it is likely that our findings are significant in the leiomyosarcoma cohort as this has the largest number of cases, but for the other tumour types, we cannot exclude that genes and DMRs identified are the result of chance variation. Due to these limitations of the sample size, further validation in expanded sample cohorts is required to establish whether these specific markers can be of prognostic significance or therapeutic targets in advanced sarcomas.

In summary, our study is the first to identify the role in sarcoma progression of differentially methylated genes (*PTPRN2* and *DAXX* in LMS; *WT1-AS* and *TNXB* in SS; *VENTX* and *NTRK3* in Ple-RMS and *MEST* and *C14MC /* miR-379/miR-656 cluster in MFS) that have been previously described in context of carcinoma progression and metastasis. Lines of further enquiry could aim to establish (1) if these potential candidate genes can be subjected to targeted therapy or (2) whether they may have utility as prognostic or predictive markers, allowing the identification of patients with sarcomas prone to develop metastasis or recurrent disease. As our cohort is enriched for sarcomas which developed early metastases and/or recurrence, it is possible that methylation for these genes may identify a subset of patients, who would benefit from more aggressive and early clinical management. In summary, we were able to demonstrate that methylation changes occur in all the sarcoma types analysed during progressive disease and appear to have an impact in the metastatic cascade.

## Supplementary information

Supplementary Information.
