# Supplementary material for: Genome wide methylation profiling of selected matched soft tissue sarcomas identifies methylation changes in metastatic and recurrent disease
Source: Sci Rep. 2021 Jan 12;11:667. doi: 10.1038/s41598-020-79648-6 (PMC7804318; doi:10.1038/s41598-020-79648-6)
Supplement: Supplementary file 1 — Supplementary Information. [file 41598_2020_79648_MOESM1_ESM.pdf]

Genome wide methylation profiling of selected matched soft tissue sarcomas identifies methylation changes in metastatic and recurrent disease

Ana Cristina Vargas*[1,2,3], Lesley-Ann Gray[4], Christine L White[5,6], Fiona M Maclean[1,2,7], Peter Grimison[3,8], Nima Mesbah Ardakani[9-10], Fiona Bonar[1], Elizabeth M Algar[5,6], Alison L Cheah[1], Peter Russell[3], Annabelle Mahar[11], Anthony J Gill [2,3,12].

1. Department of Anatomical Pathology, Douglass Hanly Moir Pathology, Macquarie Park NSW 2113 Australia.
2. Cancer Diagnosis and Pathology Group, Kolling Institute of Medical Research, Royal North Shore Hospital, St Leonards NSW 2065 Australia
3. Sydney Medical School, University of Sydney, NSW 2006 Australia.
4. Australian Genome Research Facility Ltd., Victorian Comprehensive Cancer Centre, Melbourne, VIC, 3000, Australia.
5. Genetics and Molecular Pathology Laboratory, Centre for Cancer Research, Hudson Institute of Medical Research, VIC 3168 Australia.
6. Monash University, Clayton, Victoria, Australia.
7. Department of Clinical Medicine, Faculty of Medicine and Health Sciences, Macquarie University, Sydney, NSW, Australia
8. Department of Medical Oncology, Chris O'Brien Lifehouse, Camperdown NSW 2050 Australia
9. Department of Anatomical Pathology, PathWest Laboratory Medicine, QEII Medical Centre, Perth, WA, Australia,
10. School of Pathology and Laboratory Medicine, University of Western Australia, Perth, WA, Australia.
11. Department of Tissue Pathology and Diagnostic Oncology, Royal Prince Alfred Hospital, Camperdown NSW 2050 Australia
12. NSW Health Pathology, Department of Anatomical Pathology, Royal North Shore Hospital, Sydney NSW 2065 Australia

Corresponding author

Ana Cristina Vargas

cvargas@dhm.com.au

SUPPLEMENTARY MATERIAL

SUPPLEMENTARY METHODS

1. Fluorescent in-situ hybridization.

As ancillary for histopathological diagnosis, fluorescent-in-situ hybridization (FISH) was performed at the time of the diagnosis only for synovial sarcoma and embryonal rhabdomyosarcoma (Emb RMS). FISH was performed in interphase cells using the Vysis LSI FOXO1 (13q14) Dual Color, Break Apart Rearrangement Probe for Emb RMS and the Vysis SS18 (18q11.2) Dual Color, Break Apart Rearrangement Probe (Abbott) for SS. FISH was performed following the manufacturer's instructions. Scoring of FISH hybridization signals was performed on 500 cells. Diagnostic signals for gene rearrangement demonstrated fusion of orange and green signals in >15% of the neoplastic cells.

2. FFPE DNA Extraction, Bisulphite Conversion and Restoration

DNA was extracted from at least 250mm^2 of formalin fixed paraffin embedded (FFPE) tissue using the ReliaPrep FFPE gDNA Miniprep System (Promega, Madison, WI) according to manufacturer's instructions. Extracted DNA was quantified using QuantiFluor ONE dsDNA System and Quantus instrument (Promega, Madison, WI). DNA integrity was assessed by real time PCR using the Infinium FFPE QC Kit (Illumina, San Diego, CA) and HotStar PCR reagents (Qiagen, Hilden, Germany) on a RotorGene Q (Qiagen, Hilden, Germany) instrument according to manufacturers' instructions. 500ng of DNA was bisulphite converted using the EZ DNA Methylation kit (Zymo, Irvine, CA) following the manufacturer's instructions. Bisuphite converted DNA quantity was assessed using the QuantiFluor ssDNA System and Quantus instrument (Promega, Madison, WI). The effectiveness of bisuphite conversion was assessed by methylation specific PCR for a CpG islands 76 to 87 within the *MGMT* promoter after the method of Esteller *et al.* [1]. Two separate PCR reactions to detect methylated sequence (forward primer 5'-TTTCGACGTTCGTAGGTTTTCGC-3', reverse primer 5'-GCACTCTTCCGAAAACGAAACG-3') and unmethylated sequence (forward primer 5'-TTTGTGTTTTGATGTTTGTAGGTTTTTGT-3', reverse primer 5'-AACTCCACACTCTTCCAAAAACAAAACA-3') were run using 40ng template DNA per reaction. Unmethylated and methylated reactions had the same cycling conditions (95°C 15 min followed by 35 cycles of 95°C 30 sec, 59°C 30 sec, 72°C 30 sec followed by 72°C 10 min). Infinium HD FFPE Restore kit (Illumina, San Diego, CA) was used to restore 500ng of bisulphite converted DNA.

3. Methylation array and Bioinformatics

Genome-wide DNA methylation profiling was performed using the Illumina Infinium MethylationEPIC BeadChip (Illumina, Inc., San Diego, CA, USA) using restored DNA at the Australian Genome Research Facility (AGRF). The BeadChips were scanned using an Illumina iScan. All analysis was undertaken using the R statistical environment (version 3.5.1) [2]. Quality control and probe summaries were assessed using the 'lumi' Bioconductor package [3]. The detection P values for each sample were plotted to identify any poor quality or failed samples. Probes that had failed in one or more samples (detection P values of < 0.01) were removed. Probes with SNPs at the CpG site, associated with the sex chromosomes and those shown to be cross-reactive [4] were also removed. Arrays were normalised using the SWAN normalisation and quality features re-assessed.

The degree of methylation for each probe was reported as M values, calculated as the log2 ratio of the intensities of methylated vs. unmethylated probes [5]. Methylation differences between samples were visualised on a density plot, dendrogram and through principle components analysis.

We then sought to investigate the methylation differences between primary and metastatic/recurrence tissue within each case. As tissue replicates were not feasible for this disease model we were restricted to direct comparisons of the tumour sites. A beta-value difference of >0.2 is commonly chosen to define probes with differential methylation between groups and represents the differences that can be detected at 99% accuracy [5,6]. Beta-values are defined as the ratio of the methylated probe intensity and the overall intensity with values ranging from 0 to 1, where 0 indicates unmethylated and 1 indicates fully methylated [7]. Negative values are reset to 0. The number and correlation of probes with a beta-value change of >0.2 were calculated for tissues from each case.

The DMRforPairs [8] package in R was used to identify differentially methylated regions (DMRs) of the genome. 'Regions' were defined as those with a minimum of 4 probes within a <200 bp distance of each other with a median difference in M values >1.4 between the samples of each Set. Regions were considered significant when the Benjamini Hochberg corrected p-values were <0.05. DMRs, defined as annotated Illumina categories, include TSSs (TSS1500 and TSS200), 5′-UTR (untranslated region), first exon, gene body, 3′-UTR, CpG islands, CpG islands shores, and CpG islands shelves [7].

Variance analysis was used to rank probes associated with differential methylation. The 500 probes with the largest difference in methylation were extracted from each sample. Heatmaps were generated, using M-values, for the top 50 and top 500 probes to observe the similarity

of methylation patterns among the most variable probes in each sample. The most variable probes within each set were overlapped to identify commonly variable probes/genes, which were visualized in an UpSet plot using the UpSetR package [9].

Functional analysis was undertaken using the top 500 most variable probes in each set. Pathways analysis was assessed using ReactomePA[10] and the KEGG Pathway database[11,12]. Variables genes were extracted and separated to lists of upregulated or downregulated genes based on hypo/hypermethylation status. Functional enrichment was assessed using the kegga() function in the LIMMA toolkit. [13]

4. Array CGH Method

Genomic DNA was extracted from FFPE tissue and quantified by spectrophotometry using a NanoDrop™ ND-2000 (NanoDrop, USA). Ratio of absorbance at 260/280 was used to assess DNA purity, and samples with a ratio of ~1.80 were regarded as sufficiently pure and suitable for labelling. All DNA samples were visualised on 1.0% agarose gel and fragment sizes were assessed against a 1 Kb DNA ladder. DNA was labelled using an optimised version of the protocol for ULS labelling of FFPE DNA (Agilent, Australia). Prior to labelling, reference and FFPE DNA were heat fragmented at 95°C for 10 and 2 min respectively. 250 ng of tumour and reference DNA was then chemically labelled by incubating with 0.5 μl of ULS-Cy3 and Cy5 respectively in a thermal cycler with a heated lid for 30 minutes. Cy3-Labeled tumour DNA was combined with an equal amount of Cy5-labeled sex mismatched reference DNA and samples were hybridised onto SurePrint G3 Human CGH Microarrays, 8x60K (Agilent, Australia) according to manufacturer's instructions. Following hybridisation for 40 hours, microarray slides were washed according to manufacturer's instructions and scanned on a DNA Microarray Scanner (Agilent, Australia). Scanned images were analysed by Feature Extraction software (Agilent, Australia), which normalises the fluorescent intensity of both dyes at each probe and calculates their ratio, expressed on a logarithmic scale (probe log2 ratio). It also computes a set of Quality Control (QC) metrics, including the average green and red signal intensity at all the probes as well as the background signal (noise) and signal-to-noise ratio using non-hybridising control probes. Feature Extracted Data was then analysed using CytoGenomics Software (Agilent, Australia).

REFERENCES

1 Esteller, M. *et al.* Inactivation of the DNA-repair gene MGMT and the clinical response of gliomas to alkylating agents. *N Engl J Med*. **343**, 1350-1354 (2000).

2 R Core Team. Alanguage and environment for statistical computing. R Foundation for Statistical Computing, Vienna, Austria. URL https://www.R-project.org/ (2016).

3 Huber, W. *et al.* Orchestrating high-throughput genomic analysis with Bioconductor. *Nat Methods*. **12**, 115-121 (2015).

4 Chen, Y. A. *et al.* Discovery of cross-reactive probes and polymorphic CpGs in the Illumina Infinium HumanMethylation450 microarray. *Epigenetics*. **8**, 203-209 (2013).

5 Du, P. *et al.* Comparison of Beta-value and M-value methods for quantifying methylation levels by microarray analysis. *BMC Bioinformatics*. **11**, 587 (2010).

6 Kling, T., Wenger, A., Beck, S. & Caren, H. Validation of the MethylationEPIC BeadChip for fresh-frozen and formalin-fixed paraffin-embedded tumours. *Clin Epigenetics*. **9**, 33 (2017).

7 Wang, L., Zhao, J., Li, Y., Wang, Z. & Kang, S. Genome-wide analysis of DNA methylation in endometriosis using Illumina Human Methylation 450 K BeadChips. *Mol Reprod Dev*. **86**, 491-501 (2019).

8 Rijlaarsdam, M. A., van der Zwan, Y. G., Dorssers, L. C. & Looijenga, L. H. DMRforPairs: identifying differentially methylated regions between unique samples using array based methylation profiles. *BMC Bioinformatics*. **15**, 141 (2014).

9 Conway, J. R., Lex, A. & Gehlenborg, N. UpSetR: an R package for the visualization of intersecting sets and their properties. *Bioinformatics*. **33**, 2938-2940 (2017).

10 Yu, G. & He, Q. Y. ReactomePA: an R/Bioconductor package for reactome pathway analysis and visualization. *Mol Biosyst*. **12**, 477-479 (2016).

11 Kanehisa, M. & Goto, S. KEGG: kyoto encyclopedia of genes and genomes. *Nucleic Acids Res*. **28**, 27-30 (2000).

12 Goto, S. *et al.* Organizing and computing metabolic pathway data in terms of binary relations. *Pac Symp Biocomput*. 175-186 (1997).

13 Ritchie, M. E. *et al.* limma powers differential expression analyses for RNA-sequencing and microarray studies. *Nucleic Acids Res*. **43**, e47 (2015).

SUPPLEMENTARY FIGURES:

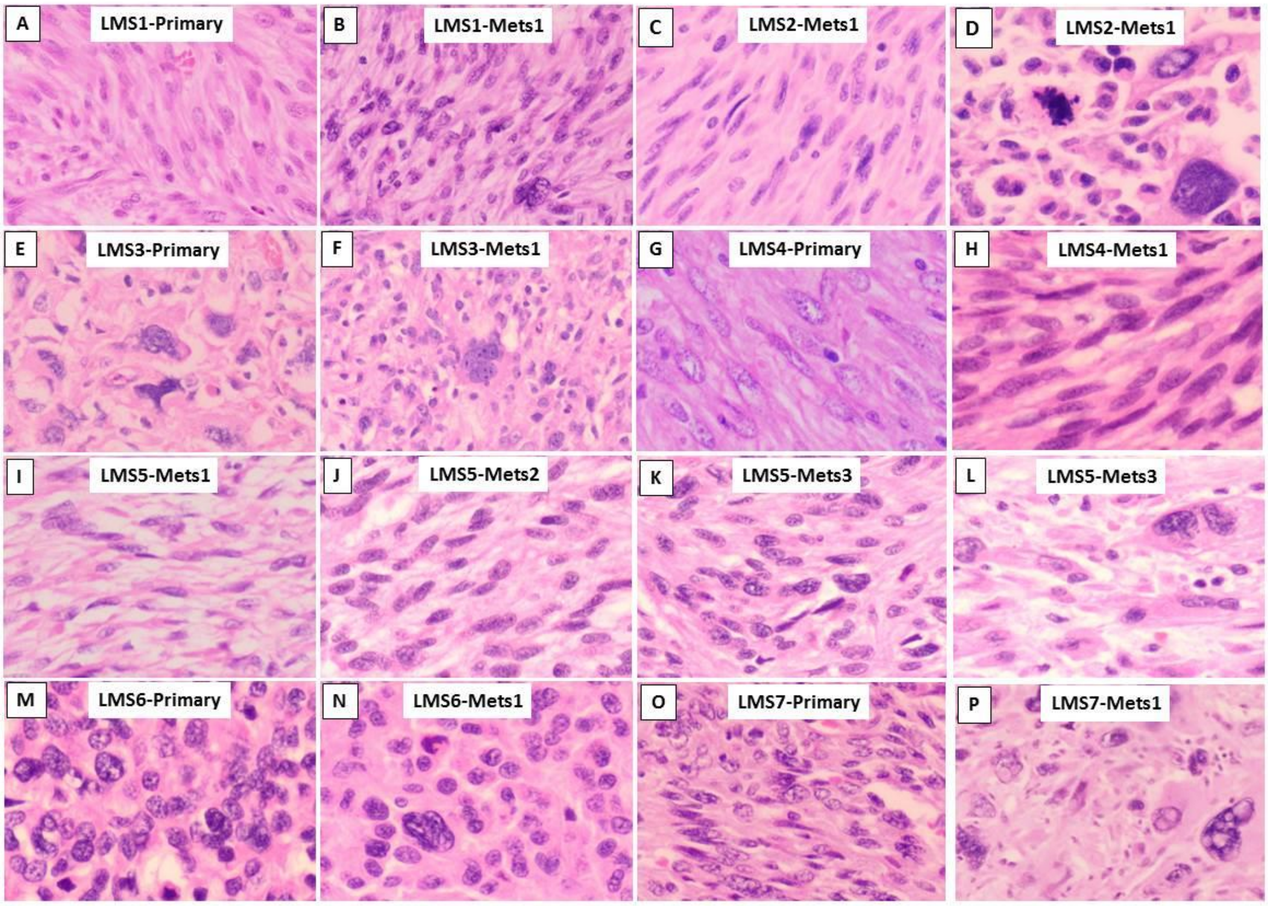

Supplementary Figure 1. Haematoxylin & Eosin-stained histological sections of leiomyosarcoma (LMS) sets. Set 1 (A-B); Set 2 (C-D); Set 3 (E-F); Set 4 (G-H); Set 5 (I-L); Set 6 (M-N) and Set 7 (O-P). Images taken at 40x.

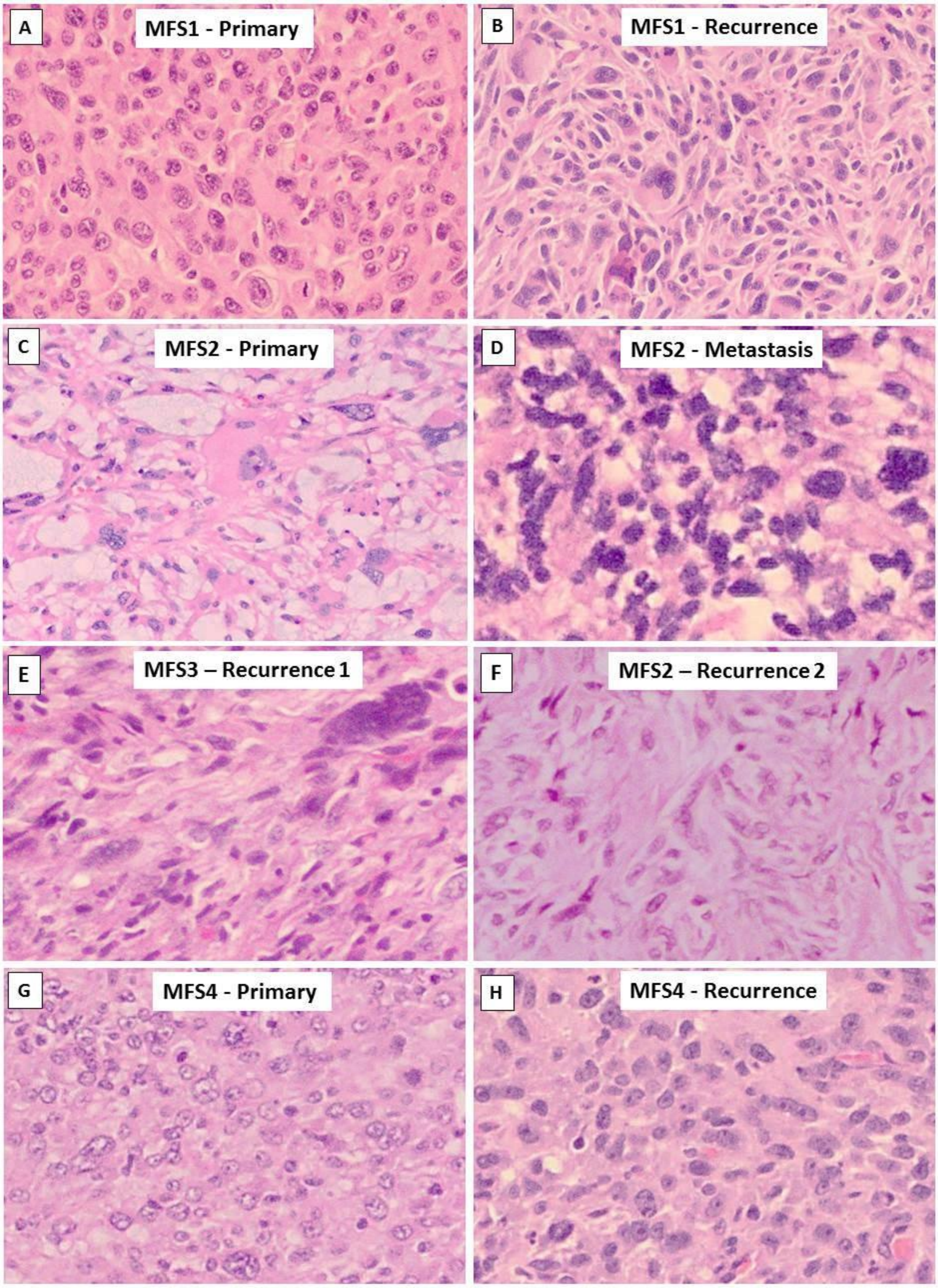

Supplementary Figure 2. Haematoxylin & Eosin-stained histological sections of myxofibrosarcoma (MFS) sets. Set 1 (A-B); Set 2 (C-D); Set 3 (E-F); Set 4 (G-H). Images taken at 40x.

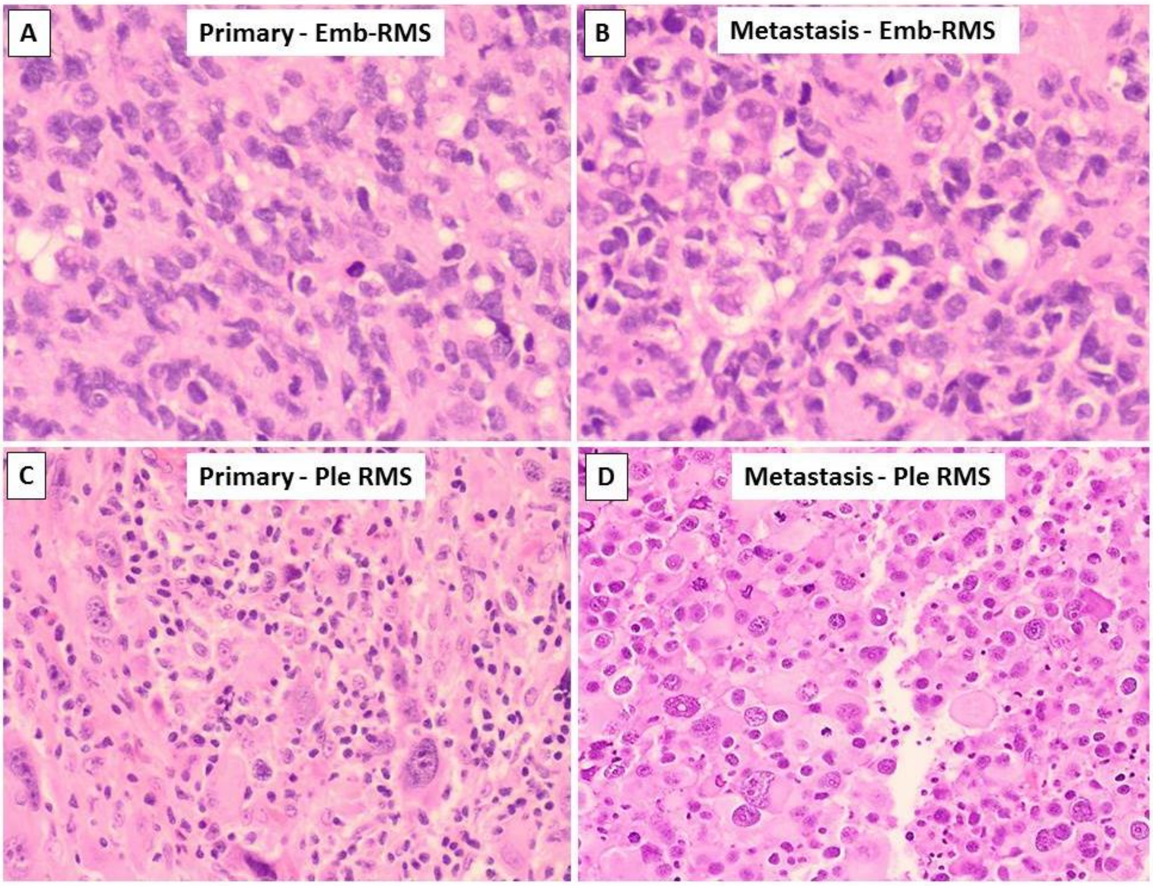

Supplementary Figure 3. Haematoxylin & Eosin-stained histological sections of embryonal rhabdomyosarcoma (EMB-RMS: A-B) and pleomorphic rhabdomyosarcoma (Ple-RMS: C-D). Images taken at 40x.

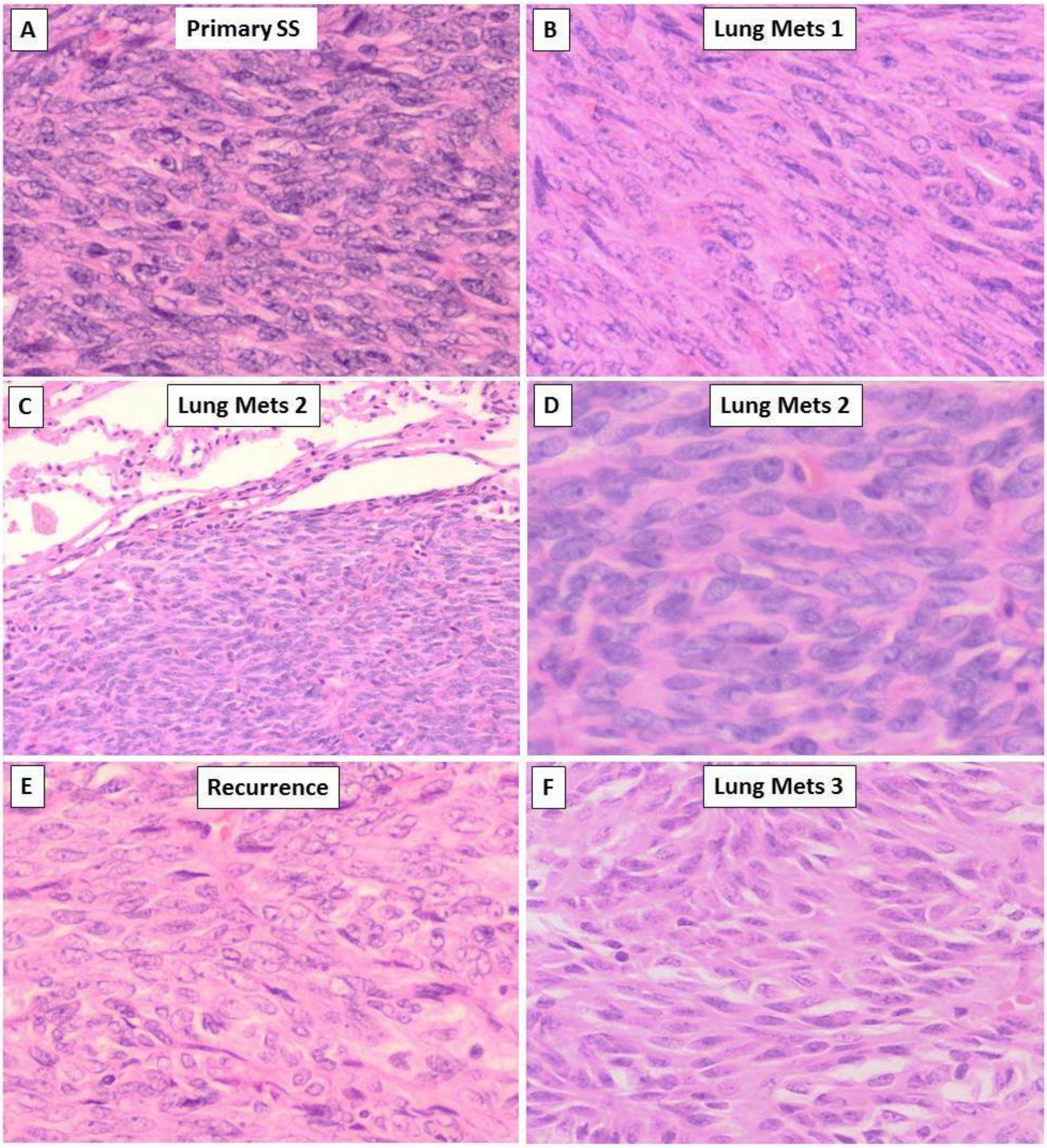

Supplementary Figure 4. Haematoxylin & Eosin-stained histological sections of all five samples derived from one synovial sarcoma (SS) patient used for the analysis. Images taken at 40x.

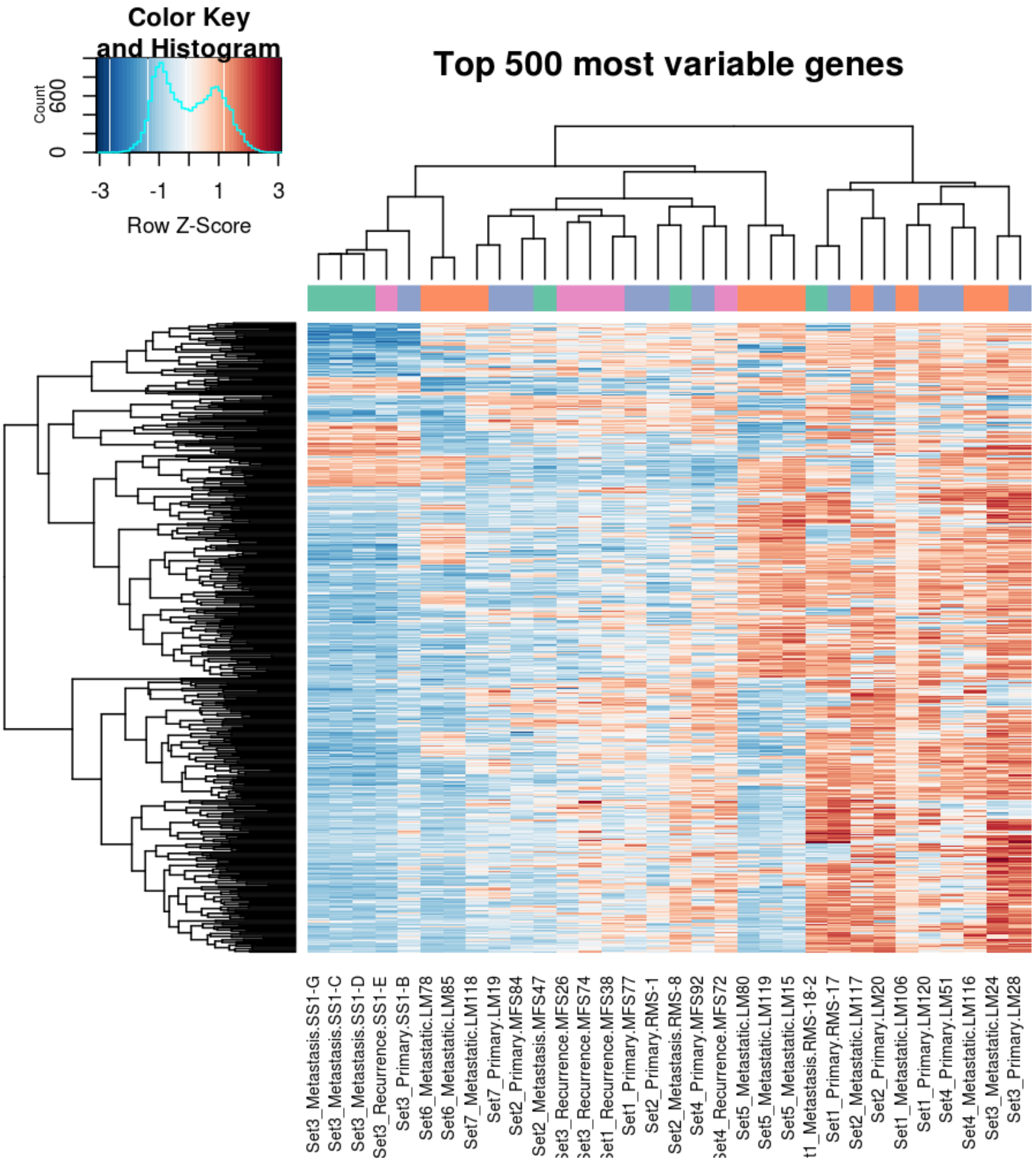

Supplementary Figure 5. Heatmap of the top 500 most variable probes across the samples using minimal filtering (P<0.01). The rows are clustered hierarchically by probe similarities and columns are clustered according to sample similarities. Leiomyosarcomas (LM); Myxofibrosarcomas (MFS); Synovial sarcoma (SS1); Embryonal (Emb) rhabdomyosarcomas (RMS) is referred to as Set 1 RMS and Pleomorphic (Ple) RMS is referred to as Set 2 RMS. Red and blue represents hypermethylation and hypomethylation, respectively. Clustering heat maps created using M values. Illumina Infinium MethylationEPIC BeadChip and R statistical environment (v.3.5.1).

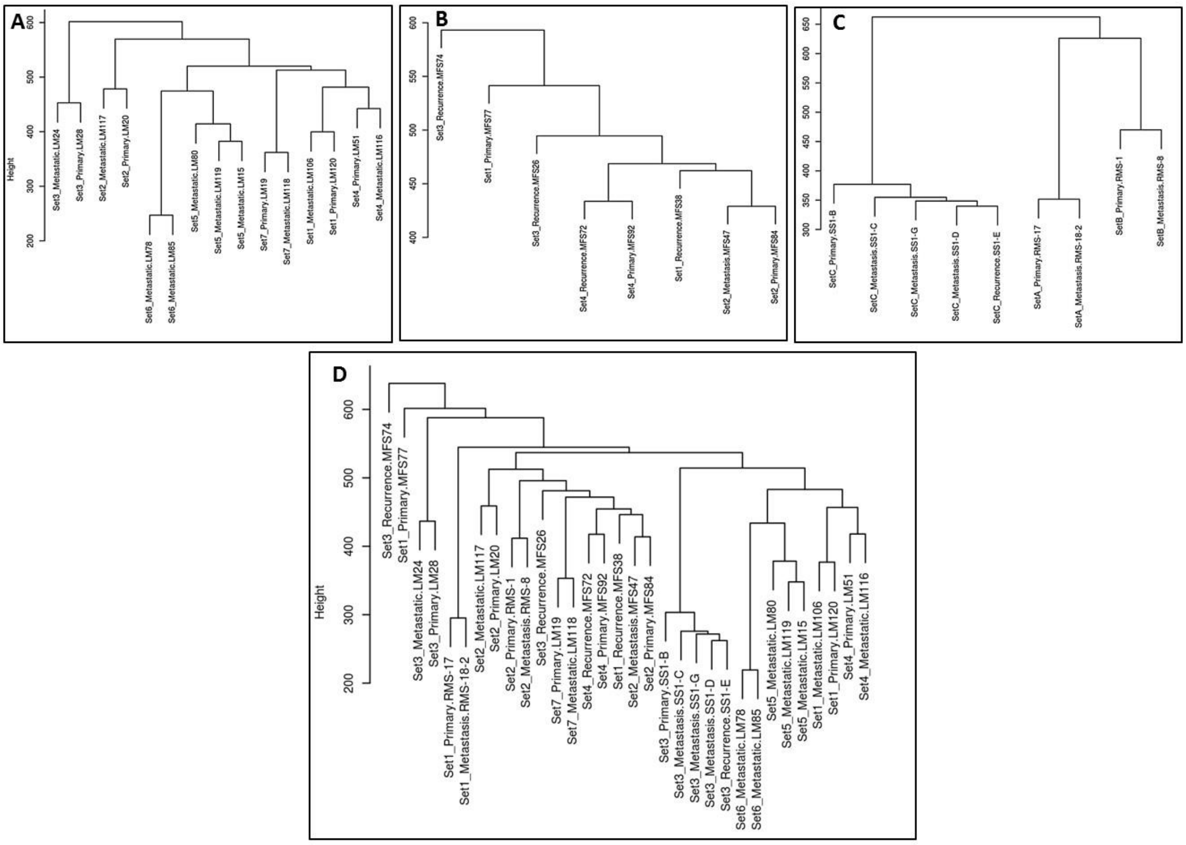

Supplementary Figure 6. Dendograms of sample relations generated according to tumour type: Leiomyosarcomas (LM: A), Myxofibrosarcomas (MFS: B) and synovial sarcoma and rhabdomyosarcomas (SS/RMS: C). Combined dendogram with all samples (D). Illumina Infinium MethylationEPIC BeadChip and R statistical environment (v.3.5.1).

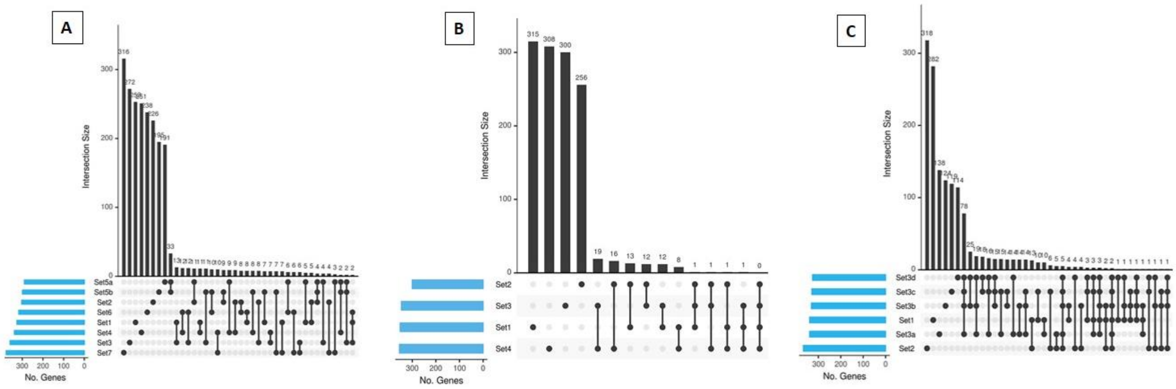

Supplementary Figure 7. UpSet plot showing the intersections of genes that were commonly among the 500 most variable probes in each sample. Data highlights that the majority of

variation is being driven by genes unique to each sample. A. Leiomyosarcoma sets; B. Myxofibrosarcoma sets; c. Synovial sarcoma and rhabdomyosarcomas. Illumina Infinium MethylationEPIC BeadChip and R statistical environment (v.3.5.1).

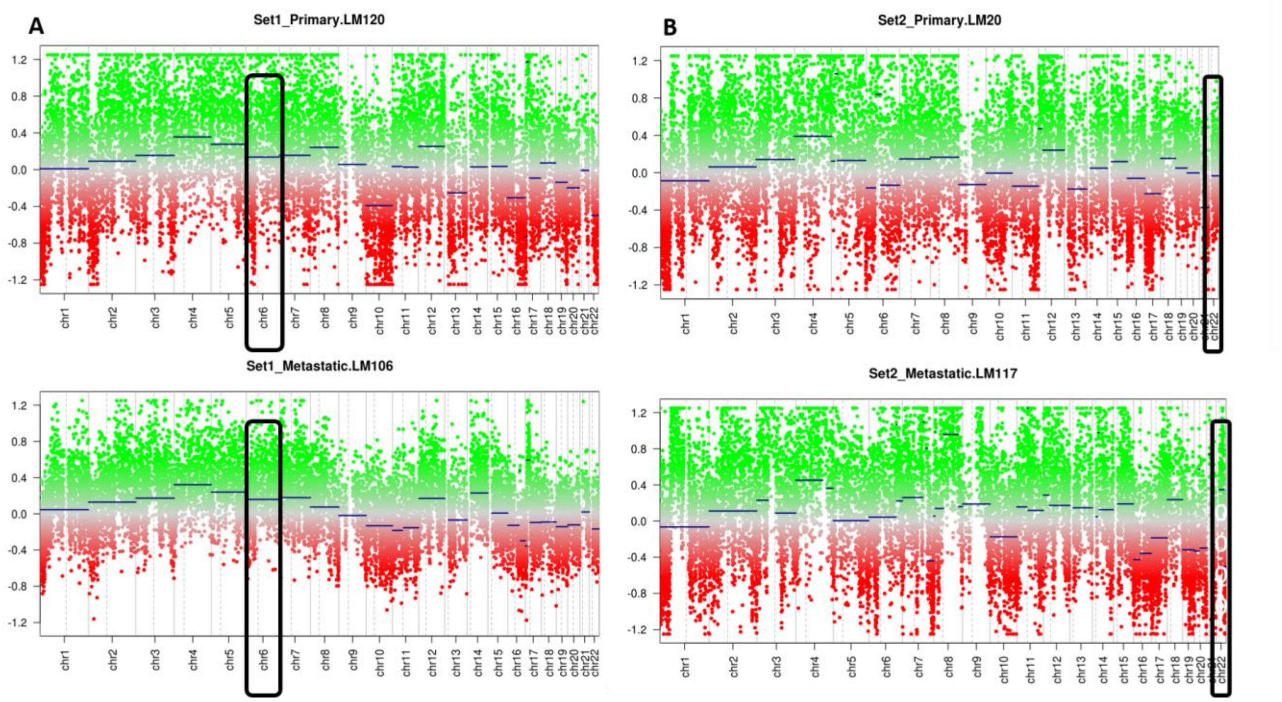

Supplementary Figure 8. Copy number variant (CNV) plots for Leiomyosarcoma (LMS) Sets 1 (A: top and bottom panel) & 2 (B: top and bottom panel) calculated using the program Conumee in R as described in methods. The CNV calling algorithm (DNACopy) establishes a threshold of <-0.3 and 0.3 for copy loss and gain, respectively. Chromosomes highlighted represent location of differentially methylated regions (DMRs).

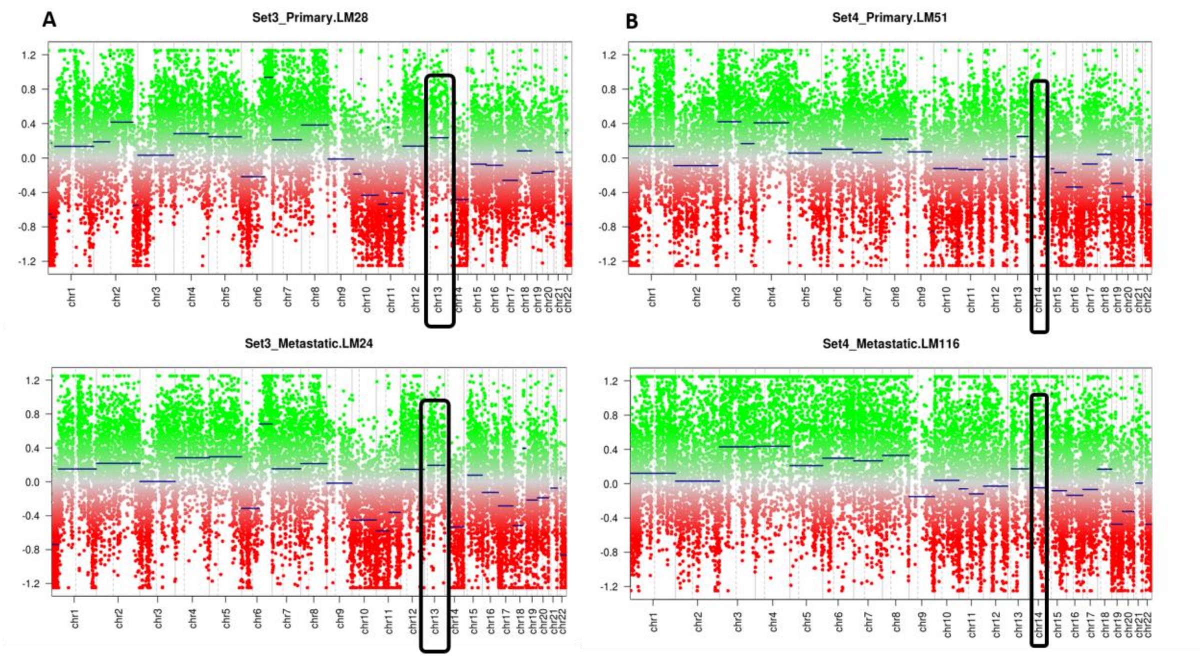

Supplementary Figure 9. Copy number variant (CNV) plots for Leiomyosarcoma (LMS) Sets 3 (A: top and bottom panel) & 4 (B: top and bottom panel) calculated using the program Conumee in R as described in methods. The CNV calling algorithm (DNACopy) establishes a threshold of <-0.3 and 0.3 for copy loss and gain, respectively. Chromosomes highlighted represent location of differentially methylated regions (DMRs).

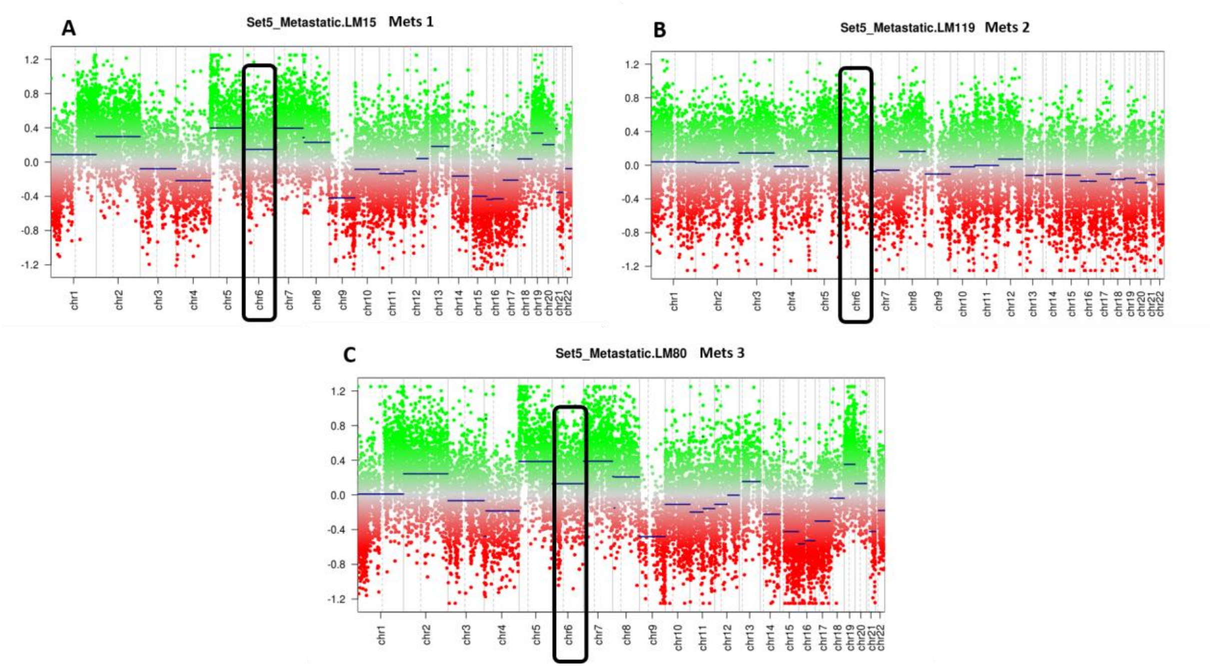

Supplementary Figure 10. Copy number variant (CNV) plots for Leiomyosarcoma (LMS) Set 5 for Metastatic (Mets) deposit 1 (A), Mets2 (B) & Mets 3 calculated using the program Conumee in R as described in methods. The CNV calling algorithm (DNACopy) establishes a

threshold of <-0.3 and 0.3 for copy loss and gain, respectively. Chromosomes highlighted represent location of differentially methylated regions (DMRs).

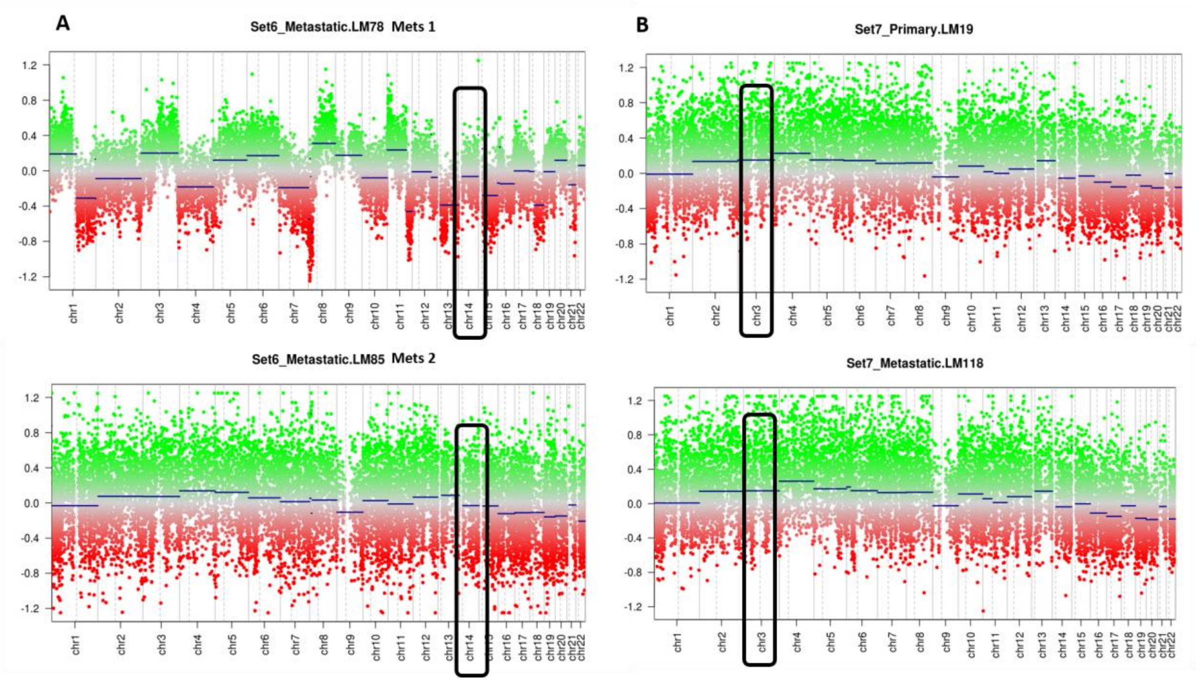

Supplementary Figure 11. Copy number variant (CNV) plots for Leiomyosarcoma (LMS) Sets 6 (A: top and bottom panel) & 7 (B: top and bottom panel) calculated using the program Conumee in R as described in methods. The CNV calling algorithm (DNACopy) establishes a threshold of <-0.3 and 0.3 for copy loss and gain, respectively. Chromosomes highlighted represent location of differentially methylated regions (DMRs).

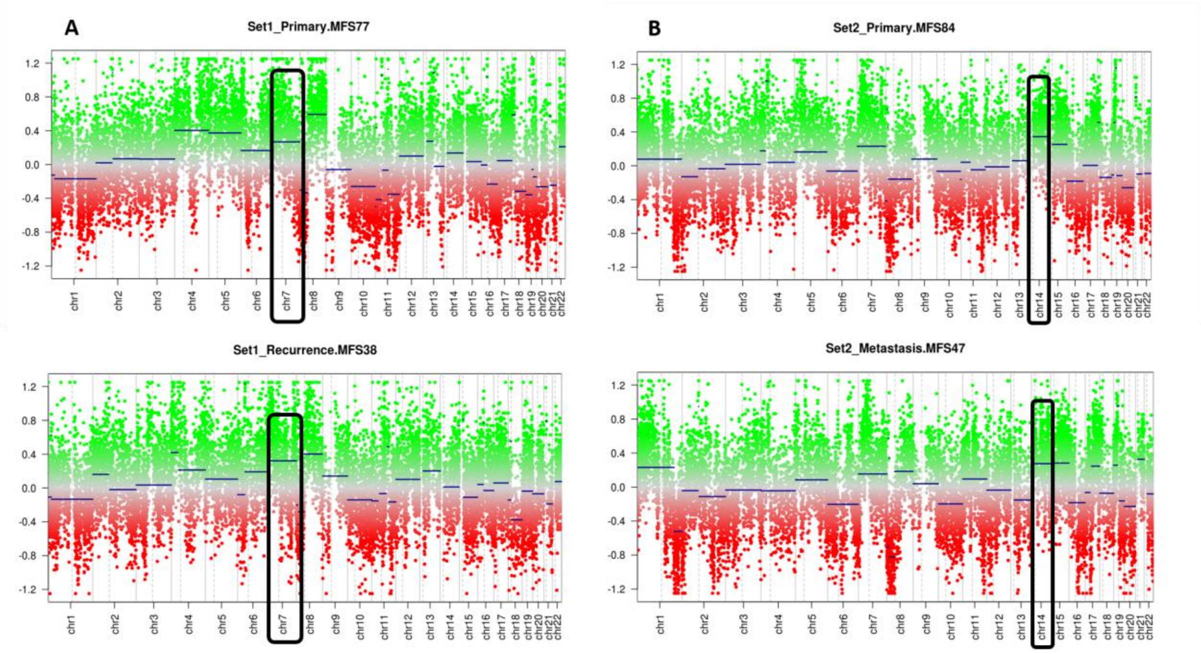

Supplementary Figure 12. Copy number variant (CNV) plots for Myxofibrosarcoma (MFS) Sets 1 (A: top and bottom panel) & 2 (B: top and bottom panel) calculated using the program Conumee in R as described in methods. The CNV calling algorithm (DNACopy) establishes a threshold of <-0.3 and 0.3 for copy loss and gain, respectively. Chromosomes highlighted represent location of differentially methylated regions (DMRs).

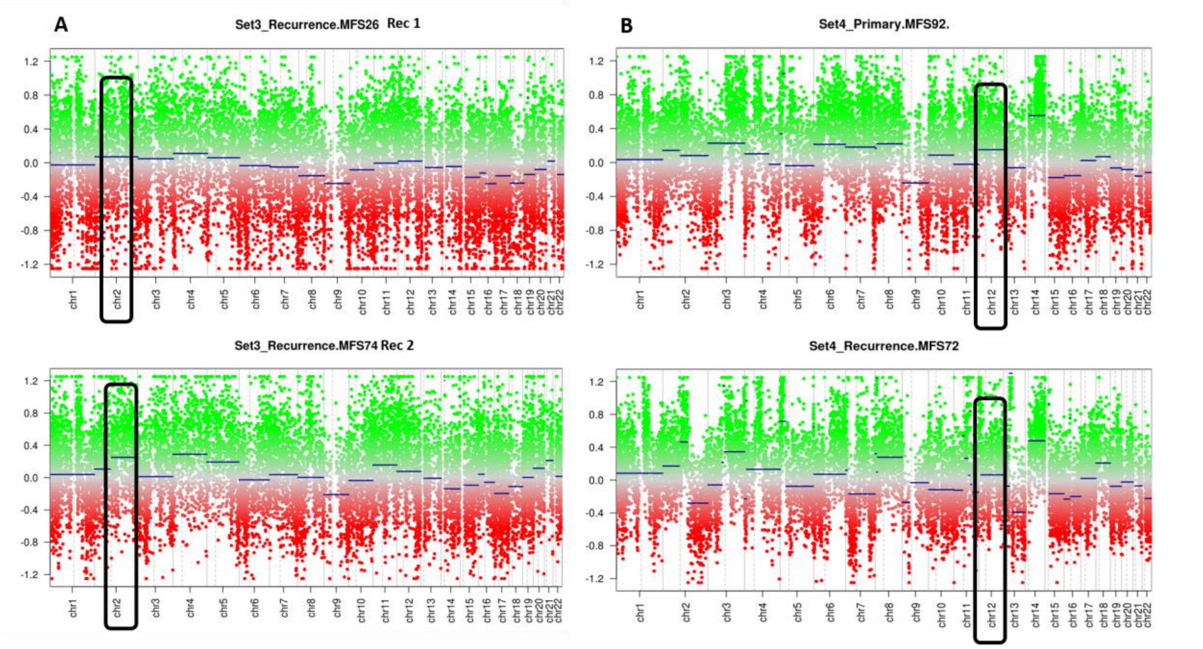

Supplementary Figure 13. Copy number variant (CNV) plots for Myxofibrosarcoma (MFS) Sets 3 (A: top and bottom panel) & 4 (B: top and bottom panel) calculated using the program Conumee in R as described in methods. The CNV calling algorithm (DNACopy) establishes a threshold of <-0.3 and 0.3 for copy loss and gain, respectively. Chromosomes highlighted represent location of differentially methylated regions (DMRs).

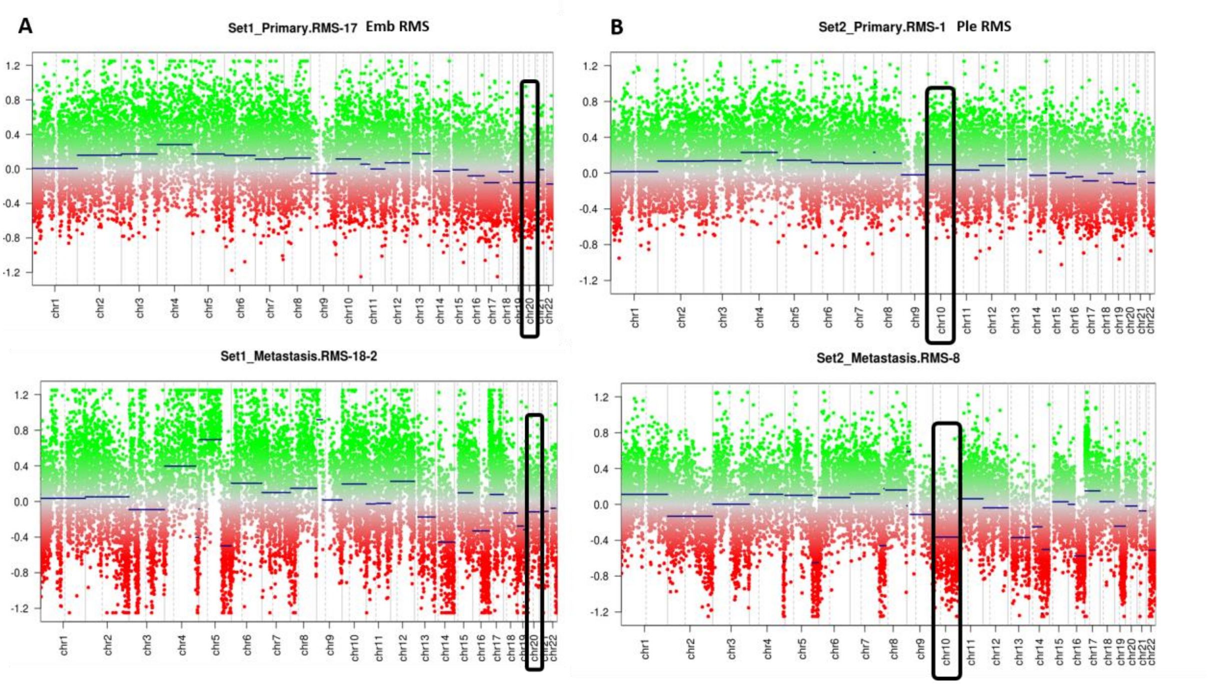

Supplementary Figure 14. Copy number variant (CNV) plots for Rhabdomyosarcoma (RMS) Sets 1/Embryonal (A: top and bottom panel) & 2/Pleomorphic (B: top and bottom panel) calculated using the program Conumee in R as described in methods. The CNV calling algorithm (DNACopy) establishes a threshold of <-0.3 and 0.3 for copy loss and gain, respectively. Chromosomes highlighted represent location of differentially methylated regions (DMRs).

Supplementary Table 1. Genes identified through Variance Analysis (Illumina Infinium MethylationEPIC BeadChip and R statistical environment (v.3.5.1))

.

Set	Probe	Chr	Chromosome location	Gene	Gene name
LMS_Set1	cg09698465	Chr 12	chr12:133000178	No genes	N/A
LMS_Set2	cg01751134	Chr 16	chr16:55543166	*LPCAT2*	Lysophosphatidylcholine Acyltransferase 2
LMS_Set3	cg15802073	Chr 16	chr16:67679091	*RLTPR/CARMIL2*	Capping Protein Regulator And Myosin 1 Linker 2
LMS_Set4	cg22849665	Chr 6	chr6:32118375	*PRRT1*	Proline Rich Transmembrane Protein 1
LMS_Set5a (M2vs.M3)	cg09639151	Chr 5	chr5: 140810920	*PCDH*	Protocadherin gene cluster
LMS_Setb (M1vs.M2)	cg21675424	Chr 22	chr22:21987039	*CCDC116*	Coiled-Coil Domain Containing 116
LMS_Set6		Chr 17	chr17: 8926794	*NTN1*	Netrin 1
LMS_Set7	cg23483707	Chr 13	chr13:44453835	*C13orf31*	Laccase domain containing 1
MFS_Set1	cg00466334	Chr 1	chr1: 119532850	*TBX15*	T-Box Transcription Factor 15
MFS_Set2	cg05559081	Chr 9	chr9: 79152490	N/A	Open sea probe on chromosome 9
MFS_Set3	cg07690326	Chr 8	chr8:145019032	*PLEC1*	Plectin-1
MFS_Set4	cg27347146			*CDH15*	Cadherin-15
SS (Prim vs. M1, M2 & M3	cg15718164	Chr 3	chr3:17364661	*TBC1D5*	TBC1 Domain Family Member 5
SS (Prim vs. Rec)	cg09450153	Chr 7	chr7: 28757771	*CREB5*	CAMP Responsive Element Binding Protein 5
Emb-RMS	cg16292451	Chr 1	chr1: 161954657	*OLFML2B*	Olfactomedin Like 2B
Ple-RMS	cg11525479	Chr 15	chr15: 88799523	*NTRK3*	Neurotrophic Receptor Tyrosine Kinase 3

Supplementary Table 2. Selected top 10 upregulated KEGG pathways[11,12] displayed per sarcoma type.

**Selected top 10 up-regulated KEGG pathways**		
**PathwayID**	**Pathway**	**P.DE**
**Leiomyosarcoma samples**		
path:hsa04024	cAMP signaling pathway*	0.003089
path:hsa04921	Oxytocin signaling pathway*	0.005397
path:hsa04022	cGMP-PKG signaling pathway	0.007337
path:hsa04010	MAPK signaling pathway*	0.012269
path:hsa04014	Ras signaling pathway	0.017575
path:hsa04350	TGF-beta signaling pathway	0.005474
path:hsa00471	D-Glutamine and D-glutamate metabolism	0.037337
path:hsa03440	Homologous recombination	0.026401
path:hsa04935	Growth hormone synthesis secretion and action	0.008499
path:hsa00534	Glycosaminoglycan biosynthesis - heparan sulfate	0.01137
path:hsa04072	Phospholipase D signaling pathway	0.017785
path:hsa04150	mTOR signaling pathway	0.001628
path:hsa04916	Melanogenesis*	0.002457
path:hsa03410	Base excision repair	0.014813
path:hsa04974	Protein digestion and absorption*	0.016351
path:hsa03030	DNA replication	0.017487
path:hsa05205	Proteoglycans in cancer	0.027676
path:hsa04512	ECM-receptor interaction	0.031362
path:hsa04658	Th1 and Th2 cell differentiation	0.035115
**Myxofibrosarcoma samples**		
path:hsa04024	cAMP signaling pathway	6.59E-03
path:hsa04060	Cytokine-cytokine_receptor_interaction	1.46E-02
path:hsa04512	ECM-receptor interaction	4.25E-02
path:hsa00512	Mucin type O-glycan byosinthesis	3.03E-02
path:hsa00350	Tyrosine metabolism	3.58E-02
path:hsa04020	Calcium signaling pathway	1.38E-02
**Synovial sarcoma**		
path:hsa04020	Calcium signaling pathway	4.70E-02
path:hsa04725	Cholinergic synapse	2.70E-04
path:hsa04911	Insulin secretion	2.70E-03
path:hsa04925	Aldosterone synthesis and secretion	3.90E-03
path:hsa04927	Cortisol synthesis and secretion	3.30E-06
path:hsa04940	Type I diabetes mellitus	3.70E-02
**Embryonal rhabdomyosarcoma**		
path:hsa03320	PPAR signaling pathway	1.00E-02
path:hsa03030	DNA replication	1.90E-02
path:hsa03440	Homologous recombination	2.40E-02
path:hsa04670	Leukocyte transendothelial migration	2.90E-02
**Pleomorphic Rhabdomyosarcoma**		
path:hsa04020	Calcium signaling pathway cAMP signaling pathway	3.30E-05
path:hsa05200	Pathways in cancer	4.00E-05
path:hsa04550	Signaling pathways regulating pluripotency of stem cells	4.80E-05
path:hsa04024	cAMP signaling pathway	9.90E-05

Pathways analysis assessed using ReactomePA ,the Reactome Homo sapiens database and KEGG pathway analysis [11,12].Please note that only selected pathways differentially identified in paired analysis and within the Top10 are displayed in this table. Overlapped pathways (only if within the op10) are indicated with an asterisks.

Supplementary Table 3. Selected top 10 downregulated KEGG pathways[11,12] displayed per sarcoma type.

Selected top 10 down-regulated KEGG pathways		
**PathwayID**	**Pathway**	**P.DE**
**Leiomyosarcoma samples**		
path:hsa04350	TGF-beta signaling pathway	0.02858
path:hsa04512	ECM-receptor interaction	0.001501
path:hsa03020	RNA polymerase	0.00131
path:hsa03050	Proteasome	0.040083
path:hsa04510	Focal adhesion	0.025868
path:hsa04340	Hedgehog signaling pathway	0.032573
path:hsa04961	Endocrine and factor-regulated calcium reabsorption	0.005526
path:hsa04662	B cell receptor signaling pathway	0.01819
path:hsa04020	Calcium signaling pathway	5.20E-06
path:hsa04911	Insulin secretion	0.002716
path:hsa03320	PPAR signaling pathway	0.048478
path:hsa04150	mTOR signaling pathway*	0.008221
path:hsa04010	MAPK signaling pathway	0.01844
**Myxofibrosarcoma samples**		
path:hsa00532	Glycosaminoglycan biosynthesis	5.61E-03
path:hsa04350	TGF-beta signaling pathway	1.60E-02
path:hsa03030	DNA replication	1.76E-02
path:hsa04660	T cell receptor signaling pathway	2.09E-02
path:hsa03430	Mismatch repair	2.78E-02
path:hsa04514	Cell adhesion molecules (CAMs)	2.91E-02
path:hsa04510	Focal adhesion	4.06E-04
path:hsa04151	PI3K-Akt signaling pathway	8.19E-04
path:hsa04512	ECM-receptor interaction	2.86E-03
path:hsa04014	Ras signaling pathway	6.44E-03
**Synovial sarcoma**		
path:hsa04725	Cholinergic synapse	4.50E-02
path:hsa04514	Cell adhesion molecules (CAMs)	3.30E-03
path:hsa04512	ECM-receptor interaction	3.10E-02
path:hsa04310	Wnt signaling pathway	3.60E-02
path:hsa04530	Tight junction	4.30E-02
**Embryonal rhabdomyosarcoma**		
path:hsa04024	cAMP signaling pathway	6.00E-04
path:hsa05200	Pathways in cancer	1.80E-04
**Pleomorphic Rhabdomyosarcoma**		
path:hsa04514	Cell adhesion molecules (CAMs)	3.30E-03
path:hsa04961	Endocrine and calcium reabsorption	3.30E-02

Pathways analysis assessed using ReactomePA ,the Reactome Homo sapiens database and KEGG pathway analysis [11,12]. Please note that only selected pathways differentially identified in paired analysis and within the Top10 are displayed in this table. Overlapped pathways (only if within the op10) are indicated with an asterisks.
